# Strain‐Sensitive Thermochromic Smart Electronic Skin for Joint and Spine Healthcare Applications

**DOI:** 10.1002/advs.202507605

**Published:** 2025-07-06

**Authors:** Shicheng Fan, Shuwen Chen, Zheng Qiao, Jiaming Qi, Zixiong Wu, Chwee Teck Lim

**Affiliations:** ^1^ Department of Biomedical Engineering National University of Singapore Engineering Drive 3 Singapore 117583 Singapore; ^2^ Institute of Medical Equipment Science and Engineering Huazhong University of Science and Technology Luoyu Road 1037 Wuhan 430074 China; ^3^ Institute for Health Innovation and Technology (iHealthtech) National University of Singapore 15 Kent Ridge Crescent Singapore 119276 Singapore; ^4^ Mechanobiology Institute National University of Singapore Engineering Drive 1 Singapore 117411 Singapore

**Keywords:** e‐skin, liquid metal, machine learning, motion detection, thermochromic, thermotherapy

## Abstract

Electronic skins (E‐skins) that enable interactive and quantitative joint and spine healthcare hold transformative potential for personalized musculoskeletal rehabilitation. However, existing systems face three critical limitations: conventional strain sensors degrade with cyclic large strain due to uncontrolled crack propagation; thermal therapy lacks real‐time adaptive control without feedback; and systems lack user‐interactive monitoring‐therapy integration. Here, iStretch, a mechano‐visual‐thermo adaptive e‐skin is introduced that integrates durable strain sensing, dynamic heat therapy, and intuitive visual feedback via a multiphasic and bilayer architecture. The strain‐sensing bottom layer utilises constrained microcrack propagation in graphene‐liquid metal nanocomposites, achieving high sensitivity (GF = 281), broad sensing range (300%), and exceptional durability (10,000 cycles). Simultaneously, the thermoresponsive top layer provides dynamic visual feedback and heat modulation in response to strain. Its multifunctions are demonstrated for convolutional neural network‐powered omnidirectional motion and gesture recognition, strain magnitude‐adjusted thermotherapy, intuitive thermochromic overheating alerts, and quantitative cervical spine monitoring via wireless sensor arrays.

## Graphical abstract

iStretch, a multifunctional e‐skin integrating durable strain sensing, adaptive heat therapy, and visual feedback, is presented for joint and spine rehabilitation. Featuring a bilayer graphene–liquid metal design, iStretch offers high sensitivity, real‐time responsiveness, and user interactivity, enabling personalized musculoskeletal care through motion recognition, strain‐guided thermotherapy, and wireless cervical spine monitoring.

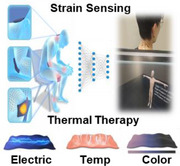

## 1 Introduction

Musculoskeletal disorders are prevalent issues that significantly impact the quality of life for individuals across various age groups, especially for the sedentary, elderly, as well as athletes suffering from cervical spondylopathy, rheumatism, tendonitis, and arthritis.^[^ ^1^, ^2^, ^3^, ^4^ ^]^ The effectiveness of rehabilitation and treatment for these conditions is highly dependent on the individual's unique biomechanical characteristics and requires long‐term therapy.^[^ ^5^ ^]^ Therefore, continuous quantitative monitoring of musculoskeletal biomechanics and providing simultaneous therapeutic interventions are essential, especially for joint and cervical spine healthcare.^[^ ^6^ ^]^

Traditional clinical tools, such as magnetic resonance imaging, offer valuable diagnostic information for joint and cervical spine healthcare but fundamentally lack continuous monitoring capabilities.^[^ ^7^ ^]^ This critical gap has driven the development of wearable electronic devices capable of both real‐time biomechanical signal acquisition^[^ ^8^, ^9^ ^]^ and therapeutic intervention.^[^ ^5^, ^10^ ^]^ Among them, electronic skin (e‐skin) technologies have emerged as promising solutions, utilizing graphenes,^[^ ^11^, ^12^, ^13^ ^]^ carbon nanotubes,^[^ ^14^, ^15^ ^]^ low dimensional metals,^[^ ^10^ ^]^ intrinsically stretchable conductors,^[^ ^16^, ^17^ ^]^ or their composites^[^ ^18^, ^19^, ^20^, ^21^, ^22^ ^]^ for strain sensing, while thermal therapy typically relies on simple ohmic heating.^[^ ^5^ ^]^ However, three fundamental challenges currently hinder their wide implementation: first, conventional strain‐sensitive nanocomposites degrade under cyclic loading due to uncontrolled crack propagation, leading to rapid signal degradation^[^ ^11^, ^12^, ^14^ ^]^ and limiting their long‐term reliability.^[^ ^11^, ^23^ ^]^ Second, existing thermal therapies operate in open‐loop mode, lacking real‐time visual feedback for adaptive heat modulation.^[^ ^18^, ^19^, ^20^, ^21^, ^22^, ^24^, ^25^ ^]^ Third, most systems fail to integrate quantitative motion tracking, interactive therapy, and intuitive user feedback into a unified platform. These limitations pose a risk of thermal burns and often lead to bulky and complex configurations, since mechanical or optical feedback often requires complicated communication^[^ ^26^, ^27^ ^]^ and display^[^ ^28^, ^29^, ^30^ ^]^ systems, integrating light emitting diodes, near field communication modules, and sensors.^[^ ^31^, ^32^, ^33^ ^]^

**Figure 1:**
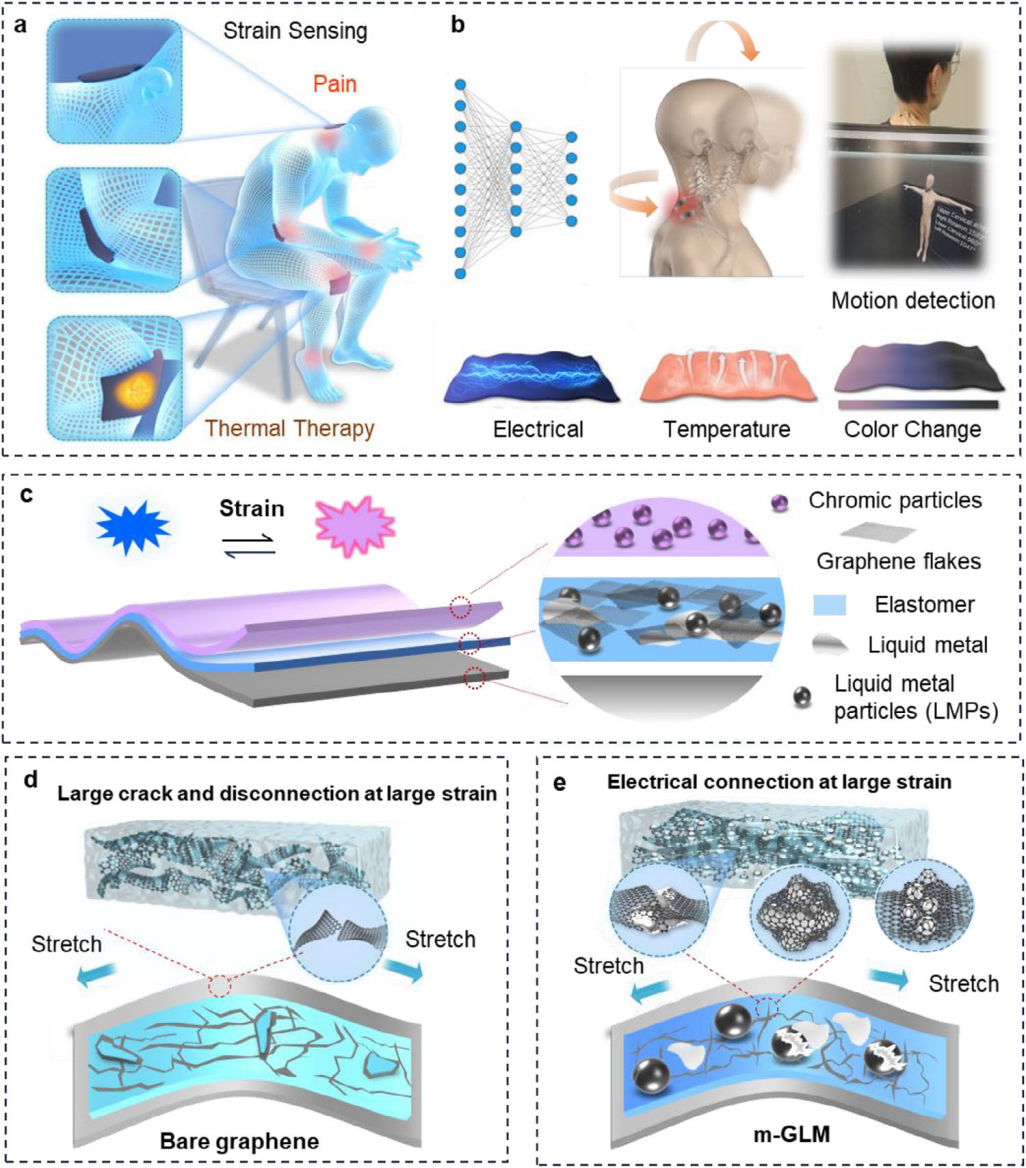
Design of the m‐GLM‐based iStretch. a) Schematic diagram of user‐interactive sensors attached to different locations of the body for strain sensing and thermal therapy with variations in electrical signal, temperature, and color changes. b) Motion identification based on 1D CNN. c) Schematic structure of the iStretch with m‐GLM composite and thermochromic materials. Illustrations of strain‐sensitive layer with d) bare graphene, and e) m‐GLM composite under stretching.

Here, we present iStretch, an intelligent strain‐adaptive thermochromic multiphasic e‐skin that uniquely integrates interactive strain sensing, adaptive thermal therapy, and real‐time visual feedback (**Figure** **1**a). The system features a compact bilayer architecture, consisting of a top thermochromic composite and bottom multiphasic composite (referred to as m‐GLM). The m‐GLM integrates three synergistic components: graphene network as the solid structural framework, bulk liquid metal (LM) as the liquid conductive medium, and dispersed LM microspheres as a core‐shell phase. This design synergistically enables continuous electrical conduction and dynamic crack‐bridging, allowing iStretch to achieve outstanding mechanical durability (>10,000 cycles), a broad strain sensing range (300%), and high sensitivity (gauge factor: 281). The thermochromic composites exhibit intuitive colorimetric responses to strain, enabling users to visually track motion intensity and receive overheating alerts. Such an iStretch enables omnidirectional musculoskeletal monitoring, capable of capturing both large joint movements and subtle physiological signals such as pulse and respiration. When coupled with a 1D convolutional neural network (CNN), it facilitates accurate recognition of complex motion patterns, significantly enhancing its applicability in personalized rehabilitation, remote monitoring, and telemedicine. We demonstrate that it can be used for joint‐bending monitoring and thermal therapy, and the e‐skin sensor arrays can be used for quantitative cervical spine assessment.

## 2 Results

### 2.1 Design and Fabrication

The intelligent e‐skin (iStretch) was designed to provide complex multifunctionality through a simple layer‐by‐layer structure and having soft, stretchable mechanical properties. Figure 1a,b shows that the iStretch can not only sense the strain for motion, gesture detection, as well as for monitoring joint conditions such as those in the cervical spine, but also can be used for thermotherapy to relieve joint pain. In addition to electrical and thermal signals, it can also illuminate optical signals to indicate induced strain and overheating. Figure 1c schematically illustrates the assembly and materials of user‐interactive strain‐sensitive iStretch (≈100 µm in thickness), in which a m‐GLM composite layer is sandwiched between a top thermochromic layer and a bottom elastomer substrate layer. The thermochromic layer is made of three kinds of thermochromic dyes and polyester polyol‐rich thermoplastic polyurethane (pp‐TPU), serving as both the visualization and package layer. The m‐GLM layer, which functions as a strain‐sensitive element, is mainly composed of Eutectic Gallium Indium (EGaIn) liquid metal particles (LMPs, Figure S1, Supporting Information), graphene nanosheets, and pp‐TPU. Unlike sensors made of solely bare graphene, which are prone to cracking, have a low strain range, and easily become unstable and dysfunctional under repeated use or large strain (Figure 1d),^[^ ^34^ ^]^ iStretch incorporates bulk liquid metal and core‐shell liquid metal particles. This combination helps to ensure high mechanical robustness and sensing stability, as shown in Figure 1e, and is also explained in detail in the Mechanism section. This property is vital for soft systems, which are easily subject to unconstrained conditions with cyclical high bending, torsion, and deformations.

The iStretch can be fabricated at scale with high resolution and customized configurations on various substrates using various printing methods (Figures S2, S3, Supporting Information). The screen‐printing method is low‐cost and easily accessible, while the desktop extrusion printer (Voltera, Figure S4, Supporting Information) is more automated and sophisticated. With the proper settings, high‐resolution traces with a minimum line width of 100 µm can be printed using nozzles with diameters of 150 or 200 µm (Figure S5a, Supporting Information). Additionally, serpentine‐shaped traces with a resolution of 200 µm and a clear edge can also be printed (Figure S5b, Supporting Information). The good printability is primarily due to the pp‐TPU binder's abundant polar groups and the use of cyclohexanone as solvent, resulting in a stable printable composite ink with low contact angles on various substrates (38.4°, 48.8°, 42.6°, and 36.5° on Glass, Very‐high‐bond (VHB) polymer, TPU, Polyethylene terephthalate substrates, respectively, as shown in Figure S6, Supporting Information). The low contact angle contributes to high work of adhesion, better wettability, and universal printability according to Young–Dupré equation (W_a_ = γ_lv_(1 + cosθ), F_w_ = γ_lv_cosθ, where W_a_ is the work of adhesion; F_w_ and θ are the wetting force and the contact angle between the ink and the substrate, respectively; γ_lv_ is the surface energy at the liquid‐vapor interface).

### 2.2 High Mechanical Resilience, Strain Sensitivity, and Wide Sensing Range

**Figure 2:**
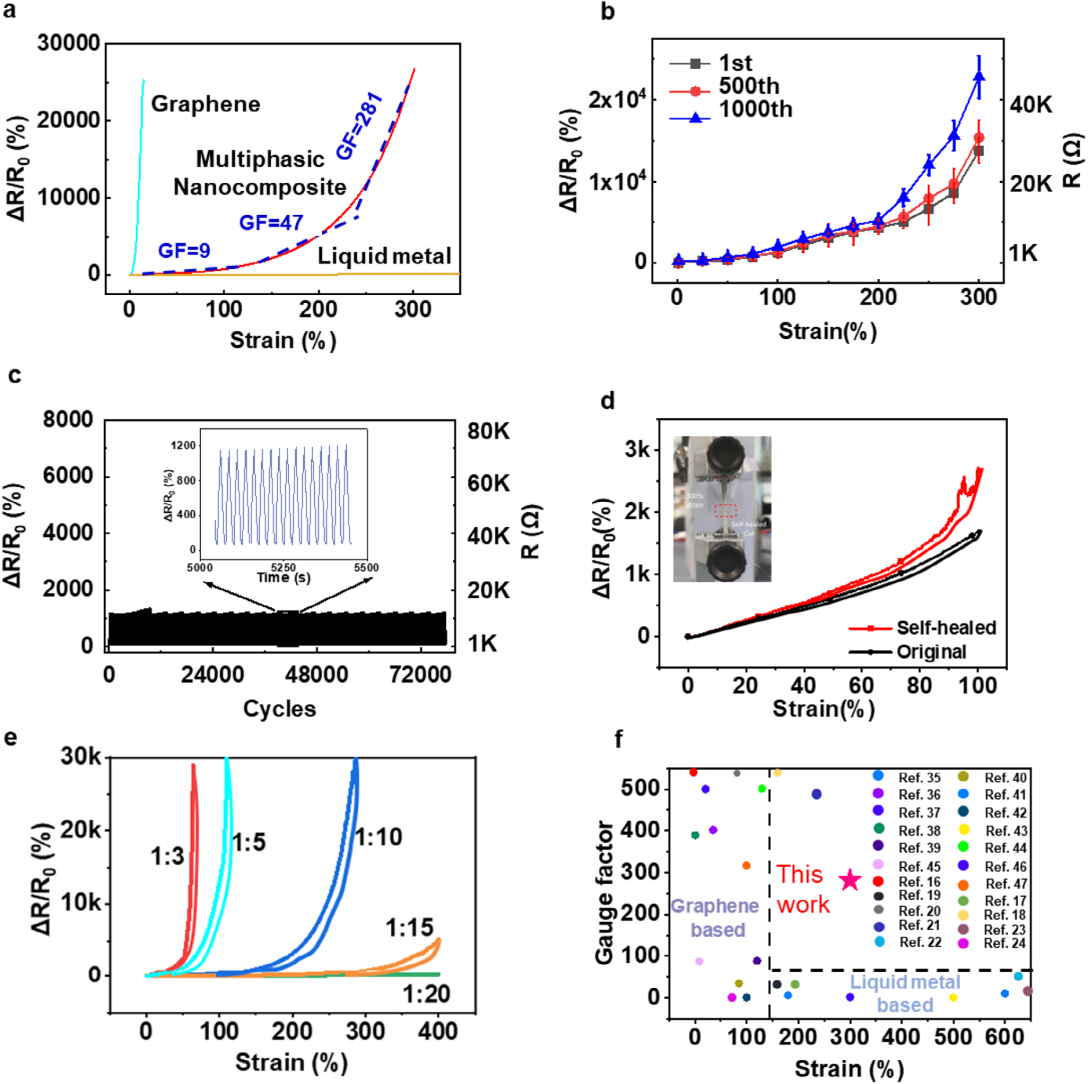
Electromechanical sensing performance. a) Relative resistance variations of strain sensors based on pure graphene (blue), pure EGaIn (yellow), and m‐GLM (red). b) Mechanical resilience of the iStretch sensor across various sample batches after different cyclical tests. c) Repeatability performance under 10000 cycles of 100% strain stretching and release with an initial resistance of ≈1000 Ω. d) A healed iStretch sensor can withstand up to 200% strain and maintain stable electromechanical performance within 100% strain. e) Resistance changes under strain for iStretch with different EGaIn/Graphene ratios. f) Comparison of the sensitivity and sensing range of recently reported strain sensors based on graphene or liquid metal.

The iStretch exhibits outstanding high‐strain sensitivity, wide sensing range, and mechanical resilience. Strain sensitivity is typically defined by gauge factor (GF) and is calculated by dividing mechanical strain by the resistance change (GF = (∆R/R_0_)/ε, where ΔR, R_0_, and ε represent the change of resistance, the original resistance, and the applied strain, respectively). As shown in **Figure** **2**a, pure graphene‐based sensors (blue curve) typically operate only within a limited stretchability range (strain <10%), while pure liquid metal‐based sensors mainly serve as conductors and exhibit low gauge factors (GF <1). In contrast, the iStretch, with a graphene and liquid metal weight ratio of 1:10, achieves GFs of 47 (110%–220% strain) and 281(220%–300%), respectively (Figure 2a; Figure S7, Supporting Information), while maintaining a GF of 9.3 for the smaller strain of 0%–110%. This performance is particularly suitable for human body interfaces, where strains typically range from 0% to 100% during activities ranging from subtle motions (speaking, blinking) to intense movements.^[^ ^35^ ^]^ The enhanced sensitivity significantly improves the signal‐to‐noise ratio, which also contributes to biomechanical strain monitoring.

To evaluate strain resilience, cyclical stretching and release tests were conducted. As shown in Figure 2b, the iStretch with TPU substrate and VHB encapsulation demonstrates nearly identical electromechanical behavior, exhibiting only 3.8% and 20.6% resistance changes after 500 and 1000 cycles at 200% strain, respectively. This strain resilience persists across both small strains (1%–50%) and relatively higher strains (50%–150%) (Figure S8, Supporting Information). Notably, the iStretch still maintains stable performance even after 10000 cycles at 100% strain, with merely 3% resistance variation (Figure 2c). However, performance degradation occurs during cycling at higher strains (220%–300%), which delineates the boundary between the elastic region (0%–220% strain) and non‐elastic region (220‐300% strain) as shown in Figure S7b (Supporting Information). This distinct behaviour arises from different deformation mechanisms: within the elastic region, the graphene‐elastomer composite can store and release elastic potential energy, enabling complete recovery to the initial state, while in the non‐elastic region, irreversible microstructural disruptions including crack formation in the graphene network, interfacial slippage, and permanent disconnection of conductive pathways lead to a loss of electrical percolation and consequent higher baseline resistance.

This strain resilience can also be enhanced by the healibity of the iStretch sensor with VHB substrates or encapsulations. As shown in Figure 2d and Figure S9, Video S1 (Supporting Information), a healed sensor can withstand up to 200% mechanical strain and maintain stable electromechanical performance within 100% strain. We infer that such healability depends on two aspects: first, self‐healability of VHB supporting layers can help re‐adhere two separated parts together and recover physical support; second, adjacent separated liquid metal pathways can self‐merge to help fill the cutting breakage and help iStretch maintain the electromechanical performance. Due to the high mechanical resilience, the iStretch can be expected to have a longer lifespan.

Furthermore, the sensitivity and sensing range can also be customized by adjusting the composite ratio (Figure 2e). For example, the iStretch sensor with a graphene‐to‐liquid metal ratio of 1:5 shows GFs of 35 and 509 under strains of 0%–50% and 70%–90%, respectively. Increasing the graphene‐to‐liquid‐metal ratio to 1:3 further increases the GF to 77 and 3400 under strain of 0%–40% and 54%–64%, respectively. Conversely, reducing the ratio to 1:15 increases the sensing range to more than 400, but the GF becomes quite small. These results suggest that adding graphene increases the GF but reduces the sensing range, while adding EGaIn broadens the sensing range but reduces sensitivity. Considering the balance of strain resilience, sensitivity, and sensing range, the graphene‐to‐liquid‐metal weight ratio of 1:10 was chosen unless otherwise specified.

To provide a more comprehensive overview, we compared our iStretch with recently reported sensors. As shown in Figure 2f and Table S1 (Supporting Information), most reported graphene‐based sensors are limited to a strain sensing range of less than 100%, while LM‐based sensors typically exhibit GFs less than 10.^[^ ^5^, ^36^, ^37^, ^38^, ^39^, ^40^, ^41^, ^42^, ^43^, ^44^, ^45^, ^46^, ^47^ ^]^ For example, a novel flexible aquatic tactile sensor based on a waterproof graphene (GR)/carbon nanotube (CNT)/polydimethylsiloxane (PDMS) composite was reported, achieving a strain sensing range of up to 128% with an exceptionally high gauge factor (GF) of 2296. However, due to the inherent brittleness of CNT and GR networks, the mechanical robustness of the composite was limited, thereby restricting the strain range of CNT/GR‐based strain sensors.^[^ ^48^ ^]^ In contrast, patterning liquid metal (LM) and copper composite electrodes (LM@Cu) on flexible substrates was developed to achieve a significantly broader sensing range of up to 600% strain, with a maximum GF of 47.^[^ ^49^ ^]^ Nevertheless, the fluidic nature of the liquid metal reduced the sensitivity of the composite. Additionally, an LM–graphene hybrid approach was also explored using a sponge‐inspired porous hydrogel composed of poly(acrylic acid), reduced graphene oxide, and 20% LM (PAA–LM20/rGO‐25). This design enabled multi‐mode sensing and excellent stretchability.^[^ ^45^ ^]^ However, despite its high extensibility, the GF remained relatively modest at 9.86 even when the applied strain exceeded 600%, highlighting a trade‐off between sensitivity and stretchability in such hybrid systems.

In contrast, our iStretch sensor exhibits both a large working range and a relatively high GF. Additionally, the sensor demonstrates rapid response characteristics (less than 100 ms, Figure S10, Supporting Information), enabling the detection of rapid frequency strain changes. This unique combination of attributes addresses the longstanding trade‐off between sensing range, mechanical resilience, and sensitivity that has hindered widespread adoption of e‐skin strain sensors. We attribute these superior comprehensive performance characteristics to the synergistic interaction between EGaIn and graphene during stretching, which will be discussed later.

### 2.3 Mechanisms for the High Electromechanical Performance

**Figure 3:**
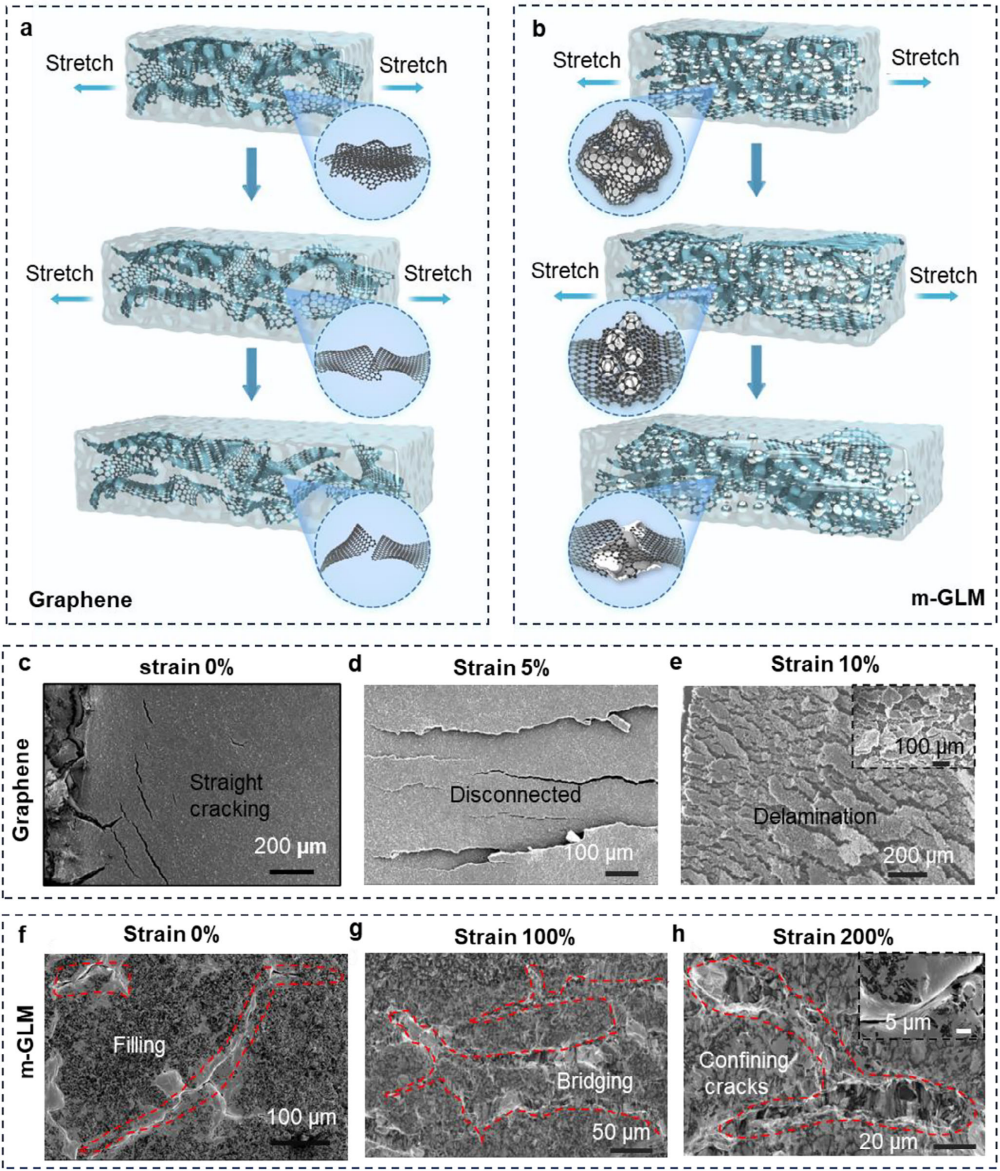
Schematic illustration of sensing mechanisms for a) pure graphene, and b) m‐GLM‐based strain sensor under stretching. The SEM of pure graphene sensing films under c) 0%, d) 5%, and e) 10% strain. f–h) The SEM images of the m‐GLM composite layer under f) 0%, g) 100%, and h) 200% strain.

We hypothesize that the outstanding mechanical resilience, wide sensing range, and high sensitivity of the iStretch sensor arise primarily from its multiphasic architecture, which integrates a solid graphene network, bulk liquid metal (EGaIn), and dispersed core–shell EGaIn microspheres. In contrast to conventional graphene‐based sensors—where weak *π*–*π* stacking between graphene sheets and the brittleness of the nanosheets cause interlayer delamination and crack propagation under strain (**Figure** **3**a),^[^ ^50^ ^]^ elongation can cause delamination of the stacked graphene layers and the fracture of graphene nanosheets, as shown in Figure 3a. This coupling of disconnection and crack propagation^[^ ^51^ ^]^ leads to the sabotage of conductive channels, resulting in a rapid increase in resistance. To address these limitations, we embedded liquid metal into the graphene network to form a multiphasic structure that modulates deformation behavior through three primary mechanisms (Figure 3b): 1) bulk EGaIn acts as a conductive, flowable matrix that bridges cracks and gaps to preserve electrical continuity; 2) strong interfacial interactions between graphene and EGaIn prevent sheet separation, reinforcing mechanical stability; and 3) core–shell EGaIn particles act as lubricating agents, introducing rolling friction to reduce shear stress and slow crack propagation. Together, these synergistic mechanisms within the multiphasic structure enable the iStretch to achieve exceptional electromechanical performance under extreme and repeated deformation.

To validate this hypothesis, both pure graphene and m‐GLM sensing layers under stretching conditions were characterized by SEM. In the pure graphene sensing layer, distinguishable gaps were observed in the intact condition (Figure 3c). As the tensile strain increased from 5% to 10%, the gaps widened, the number of cracks increased, and the length of cracks propagated (Figure 3d). These ultimately result in macro gaps, debonding of fragments, and complete fracture of the network (Figure 3e). The increase in cracks in the numbers, width, and length, as well as the exfoliation, led to a significant increase in tunnelling resistance and unrecoverable morphological damage, causing high GF, narrow strain ranges, and cyclical instability. Comparatively, the biphasic nanocomposite layer exhibits fewer macroscopical cracks under higher strain (Figure 3f–h). Figure 3f shows that the m‐GLM layer after activation with 0% strain exhibits microcracks filled by bulk liquid metal. When stretched to 100% strain, the cracks widen in pure graphene films, but most of them are filled with and remain connected by bulk liquid metal (Figure 3g). With further stretching to 200% strain, the width of surface gaps increases, but the liquid metal between cracks stretches as well, maintaining connections beneath cracks (Figure 3h; Figure S11, Supporting Information). No exfoliation or large gaps are observed. These results suggest that the presence of stretchable bulk EGaIn helps limit the sharp growth of cracks in terms of numbers, width, and length, contributing to the strain resilience and large strain sensing range.

In addition, the small EGaIn micro/nano spheres scattered throughout the network (Figure S12, Supporting Information) can help to regulate the mechanical behaviors of graphene nanosheets through a strong graphene‐EGaIn‐graphene interface. According to the Johnson, Kendall, and Robert's theory,^[^ ^52^ ^]^ the adhesive force between two material surfaces (*F_FKR_*) can be calculated by:

(1) $$F_{FKR}=E*\left[\frac{4}{3}\frac{a^{3}}{R}-{\left(\frac{8W_{AB}\pi a^{3}}{E^{*}}\right)}^{1/2}\right]$$

where $E^{*}=\frac{E}{1-\vartheta^{2}}\;$, E is the Young's modulus of elasticity, ϑ is Poisson's ratio, *R* is the sphere radius, and *W_AB_* is the work of adhesion between surface A and surface B. *W_AB_* can be simply expressed with the following equation^[^ ^53^ ^]^:

(2) $$W_{AB}=2{\left(\gamma^{A}\gamma^{B}\right)}^{1/2}$$

where γ^*A*^ and γ^*B*^ are the surface energies for materials A and B, respectively. Based on the above equations, we can estimate the work of adhesion at the interfaces of graphene‐graphene (W_G‐G_) and EGaIn‐graphene (W_EGaIn‐G_). It was reported that the surface energies of graphene and EGaIn are 46.7 and 624 mJ/m^2^, respectively.^[^ ^54^ ^]^ The dispersive work of interfacial adhesion for each type of interface can be calculated as W_G − G_ =  93.4 mJ/m^2^ and W_EGaIn − G_ =  341 mJ/m^2^. The increased work of interfacial adhesion indicates that the presence of EGaIn enhances the connection and adhesion between two graphene layers, providing an anchor function that significantly reduces the slippage between adjacent layers, thereby enabling them to maintain stable conductive channels across a broader strain range.

Moreover, when the strain is large enough to overcome the adhesion force, the insertion of EGaIn particles into stacked graphene layers can transform the original sliding friction between contacted graphene nanosheets into a rolling friction. According to the friction theory,^[^ ^53^ ^]^ this transformation can efficiently reduce the inner friction force between adjacent layers during slippage, facilitating layer sliding when strain is applied. This can dissipate part of the stress applied to the graphene network, reducing crack formation to a certain extent, thereby extending the strain sensing range. To illustrate this mechanism, finite element simulations of the mechanical behaviours of EGaIn particles and the graphene network under stretching were conducted using a simplified model, where several EGaIn particles are sandwiched by two graphene layers (Figure S13, Supporting Information). Under the same pulling force, the graphene network with inserted EGaIn particles exhibits more displacement than the pure graphene stacked structure, which aligns with our hypothesis.

Additionally, the core‐shell EGaIn particles can release liquid conductors under extremely high strain to bridge cracks and fill the gaps, ensuring the stability of the conductive channels and maintaining sensing ability. Generally, when graphene‐based sensors are subject to extremely high strain, irreversible destruction of conductive pathways and delamination of cracked fragments occur, resulting in the dysfunction of the sensor. However, when iStretch undergoes extreme strain, EGaIn particles tend to rupture, releasing bulk liquid metal that helps fill cracks and form new electrical connectivity, preventing irreparable damage to the sensor. In this scenario, the sensor can be reused after recalibrating the sensing curves.

### 2.4 Omnidirectional Motion Detection and Deep Learning‐Enhanced Motion Recognition

**Figure 4:**
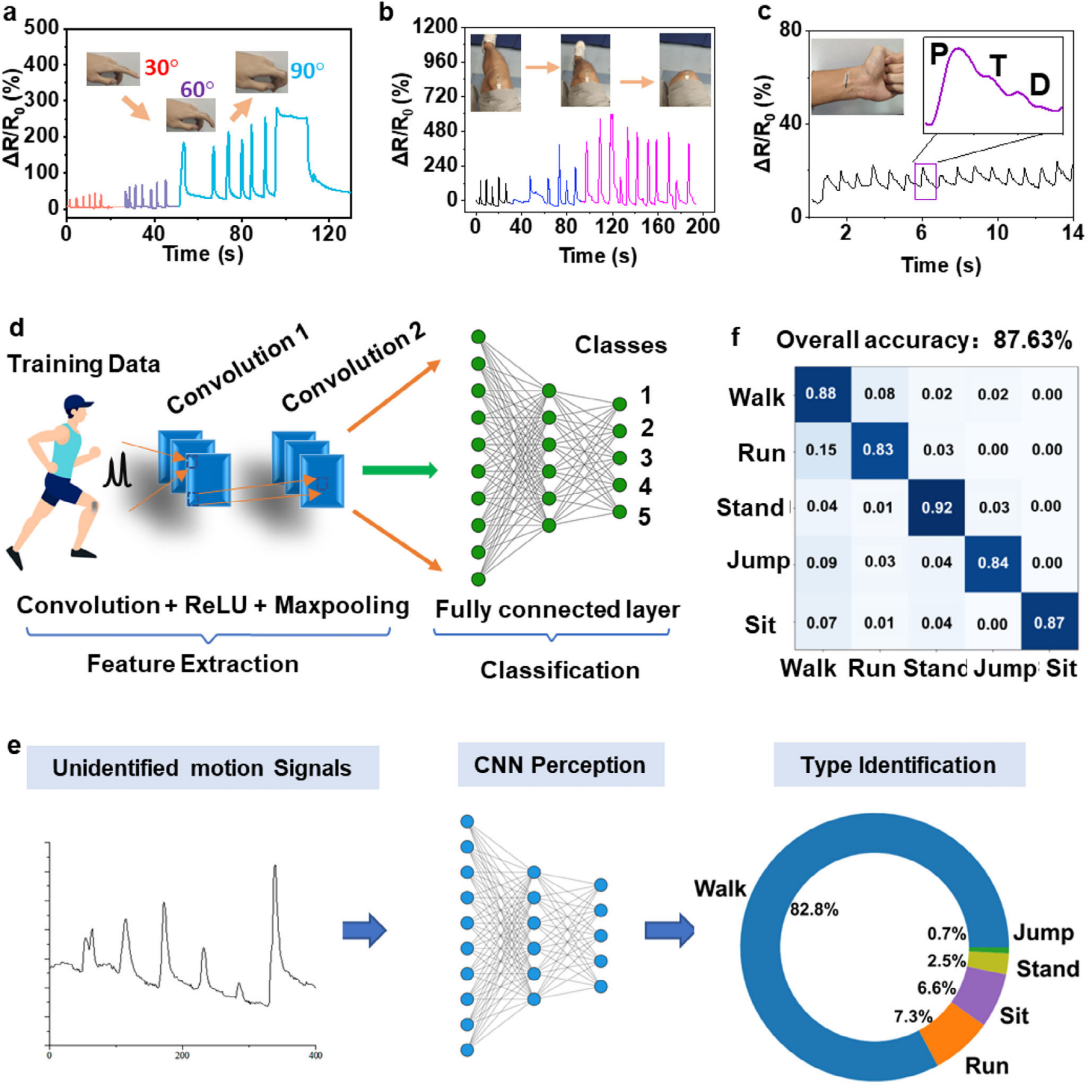
The relative resistance changes of the iStretch under a) finger bending, b) knee bending, and c) wrist pulsing. Inset in c) is the zoom‐in of a single pulse with three characteristic peaks of P‐wave, T‐wave, and D‐wave. d) A CNN model for body motion identification, comprising feature extraction, two convolutional layers, and three neuronal layers. e) A schematic illustration of a CNN perceptron for analysing an unknown input signal and identifying different types of activities with corresponding possibility distribution. f) The confusion matrix for evaluating the performance of human activity recognition performance, showing an average accuracy of 87.63%.

Taking advantage of high sensitivity and broad sensing range, we demonstrate that the sensor is capable of monitoring omnidirectional biomechanical signals, including body motions with large strains and subtle skin vibrations or fluctuations with small strains. As shown in **Figure** **4**a, the composite strain sensor, which conforms to the finger joint, can respond to different finger bending motions in real‐time, with output signals clearly distinguishing between 30°, 60°, and 90° of bending. Similarly, wrist and knee movements can also be detected (Figure S14, Supporting Information; Figure 4b), with the signal changing according to the motion's amplitude. Besides capturing large and obvious body strains, our device can also monitor subtle signals, such as muscle contractions (Figure S15a, Supporting Information), and respiratory patterns (Figure S15b, Supporting Information). Figure 4c shows that the sensor can detect stable radial arterial pulse signals from the human wrist with detailed features of three sub‐waves: the percussion wave (P‐wave), the tidal wave (T‐wave), and the diastolic wave (D‐wave). These features represent a classic radial artery pressure wave pattern, allowing for the calculation of characteristic parameters such as the radial artery augmentation index, the K value, and the time intervals,^[^ ^55^ ^]^ aiding in further analysis of radial artery health conditions and diagnosis of cardiovascular diseases.^[^ ^56^ ^]^

To demonstrate that the iStretch can recognize motions and study user behaviours for healthcare applications such as daily life‐logging, personal fitness, fall warning for the elderly, athlete training, diagnosis, and rehabilitation for motor dysfunction, we used deep learning technology to analyse the raw data recorded from the knee. Among various deep learning methods,^[^ ^57^, ^58^, ^59^ ^]^ convolutional neural network (CNN) stands out for its simple and efficient architecture, allowing it to automatically extract features and reduce data complexity. Although CNN is extensively used to process 2D image data, 1D CNN is also a useful method to extract significant features from fixed‐length segments of diverse datasets, especially for time sequences of sensor data.^[^ ^60^ ^]^ As illustrated in Figure 4d, we established a prediction model with two hidden layers, each consisting of a CNN layer, a ReLU activation function, and a Maxpooling layer. After feature extraction in the multiple hidden layers, a fully connected layer is applied to process the output signal via the SoftMax function to fulfill the final classification. The electrical signal datasets collected in the motion testing were then used to train a 1D neural network to distinguish five types of activities, including sitting, standing, walking, jumping, and running Figure S16, Supporting Information). To train and evaluate the model, we collected electrical signal datasets from motion tests conducted with the iStretch device. A total of 10 volunteers participated in the data collection process, during which each volunteer performed five distinct motion types: sitting, standing, walking, jumping, and running (Figure S16, Supporting Information). For each activity, 600 samples were recorded, yielding a dataset of 3000 samples in total.

The dataset was partitioned into training (60%), validation (20%), and testing (20%) sets. Each sample was segmented into a fixed‐length time window representing a single motion event. The model was trained over 100 epochs using the Adam optimizer, with a learning rate of 0.001 and a batch size of 32. To prevent overfitting, a dropout rate of 0.3 was applied to the fully connected layer. The network architecture featured two convolutional layers with 64 and 128 filters, respectively, each followed by ReLU activation and max‐pooling, culminating in a fully connected layer and a SoftMax output for activity classification.

Once the model is established, it can be used for motion identification in practical use. Figure 4e outlines the process flow, from inputting the sensor data to identifying the final motion type. A serial electrical signal representing an unknown motion is provided as input to the neural network. It then passes through two sequential convolution layers, and the outputs are fed into the fully connected layer, which produces the final prediction probabilities of the five different activities. The activity with the highest possibility is selected as the final output, indicating the motion type and corresponding probability. Figure 4f shows the confusion matrix for evaluating the classification accuracy by comparing the prediction labels to the true labels, with an average predicted accuracy of 87.63%.

### 2.5 Strain‐Dependent Visualized Thermotherapy

People with musculoskeletal disorders such as rheumatism, tendonitis, and arthritis often suffer from chronic pain, numbness, muscle weakness, or even a loss of athletic ability.^[^ ^61^ ^]^ Thermotherapy, which helps restore the elasticity of connective tissues and relieves tight muscles and fascia by stimulating blood circulation, is frequently used to treat this condition.^[^ ^5^ ^]^ However, joints are curvilinear and undergo a wide range of motions, and the skin can easily adapt to low‐burning temperatures, but prolonged exposure to such temperatures can easily lead to skin burns.^[^ ^62^ ^]^ To effectively rehabilitate tissues and avoid low‐temperature burns, user‐interactive stretchable heaters are required. However, current thermotherapeutic devices either use non‐stretchable heaters,^[^ ^63^ ^]^ which restrict joint motion, or only demonstrate stretchable heating without user‐interactivity.^[^ ^64^, ^65^ ^]^

**Figure 5:**
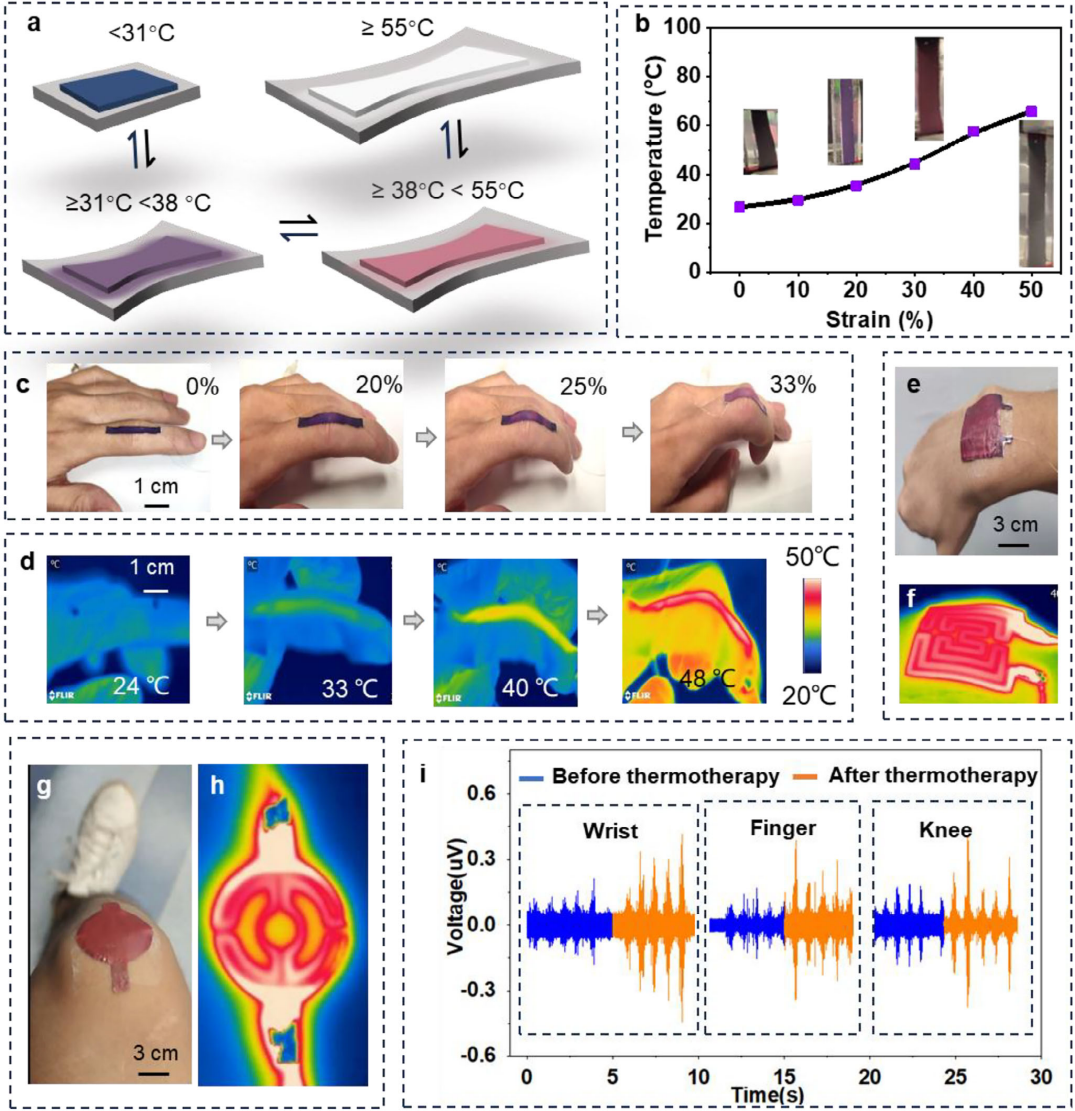
a) Illustration of the strain‐dependent color changing of iStretch. b) Temperature and color changes of iStretch as a function of tensile strain. c) Colour changes, and d) IR thermal images of an iStretch strip attached to the index finger during bending. e) The iStretch was customized to a maze shape for the wrist, and f) an IR image obtained after wrist bending. g) The iStretch was customized to a circular shape for the knee, and h) the IR image obtained after knee bending. i) EMG signals obtained from wrist, finger, and knee motions before and after thermotherapy.

Here we demonstrate that our iStretch can not only stretch to conform to curvilinear dynamic joints but also perform user‐interactive thermotherapy. We printed a thermochromic composite layer onto the strain‐sensing layer. The thermochromic composite consists of TPU elastomer mixed with green, blue, and red dyes, which turn colorless at 31, 38, and 55 °C, respectively (Figure S17a and Video S2, Supporting Information). The color changing can be briefly explained by the thermally triggered transformation of lactone rings between a low‐energy colored state and a higher‐energy colorless state.^[^ ^66^ ^]^ The mixture of the three dyes appears dark purple at a room temperature of 25 °C. As the temperature increases beyond 31, 38, and 55 °C, the composite layer transitions from dark purple to light purple, then to pink, and finally to white as the color of each dye disappears (**Figure** **5**a; Figure S17b and Video S3, Supporting Information).

To demonstrate the strain‐dependent color‐changing performance, we connected our iStretch to a power source. When a current is applied to the iStretch, thermal energy is generated (*W*  = *I* ^2^ *R*t, where *I* is the applied current, *R* is the resistance of the strain sensor, and *t* is the operating time) and diffuses to the thermochromic layer, resulting in the color change. For a fixed resistor and working time, the larger the current, the higher the temperature. As shown in Figure S20 (Supporting Information), the surface temperature gradually rises from room temperature to over 60 °C as the applied current increases from 0 to 22 mA. To characterize the visualization of color changing in response to different strains, we applied a constant current of 2 mA during stretching. Initially, no significant color change was observed due to low resistance and short operating time. As stretching continued, resistance and working time increased, leading to heat accumulation and temperature increase. The synergistic effect caused the color to quickly change from dark purple to pink, then to red, and finally to white (Figure 5b). In addition, according to previous studies,^[^ ^70^ ^,71^^]^ the variation in the resistivity of liquid metals with temperature is typically linear and relatively minor within moderate temperature ranges. Within the practical temperature range tested in our study (20–60 °C), the influence of temperature on the sensor's resistance was therefore negligible (Figure S20, Supporting Information).

Due to the generation of heat and the correlation among the strain, color, and temperature, we can attach the iStretch to different body joints for thermotherapy, visual strain sensing, and overheat warnings. Figure 5c shows that the color of iStretch changes from deep purple to purple to pink and finally to red as the figure bends, visualizing the strain changes from 0% to ≈30% and the temperature changes from room temperature to ≈40 °C. This visualized strain‐dependent sensing is also validated by the measurements of iStretch's length change and infrared (IR) thermal imaging during finger bending (Figure 5c,d). Additionally, deep pink and colorless indications can signal harmful temperatures and alert users to critical strain (Figure S19, Supporting Information). Thus, the users can then adjust the temperature and thermotherapeutic mode through intermittent joint bending and observing color changes, which helps reduce energy use, avoid overheating, and exercise the joints (Video S3, Supporting Information). Furthermore, the iStretch can be customized in different shapes and sizes to fit various irregular body joints, such as the wrist and knee (Figure 5e–h). Beyond the thermochromics, we can also incorporate a mechanoluminescent layer, serving as the user‐interactive interface. The layer can display changes in strain by varying the intensity of luminescence (Figure S20, Supporting Information).

To evaluate the impact of thermotherapeutic rehabilitation on joint function after repeated folding and unfolding and strain‐dependent heat generation, we conducted surface electromyogram (EMG) tests on the wrist, finger, and knee area. The participant was asked to perform bending cycles for 30 min (bending once and sustaining that motion of 5s) with and without the iStretch. They were also instructed to execute each motion naturally and not forcefully.^[^ ^67^, ^68^ ^]^ After 30 min of exercise, the EMG signals from different areas with iStretch exhibit a significant increase (Figure 5i). This increase suggests an expansion in the range of motion, facilitated by the heat‐induced elongation of connective tissue extensibility, which can be attributed to the effects of thermotherapy.^[^ ^69^ ^]^ We then calculated the coefficient of variation of collected EMG signals, which is defined as the ratio of the standard deviation to the mean. Generally, the coefficient of variation of EMG signals indicates data dispersion and reflects the stability of the thermotherapy effect.^[^ ^5^ ^]^ A smaller coefficient of variation suggests a lower dispersion of data and more stable thermotherapy. Figure S21 (Supporting Information) shows a smaller coefficient of variation post‐thermotherapy, which indicates the efficacy in stable thermotherapy of our iStretch.

### 2.6 Sensor Array for Remote Cervical Spine Monitoring

**Figure 6:**
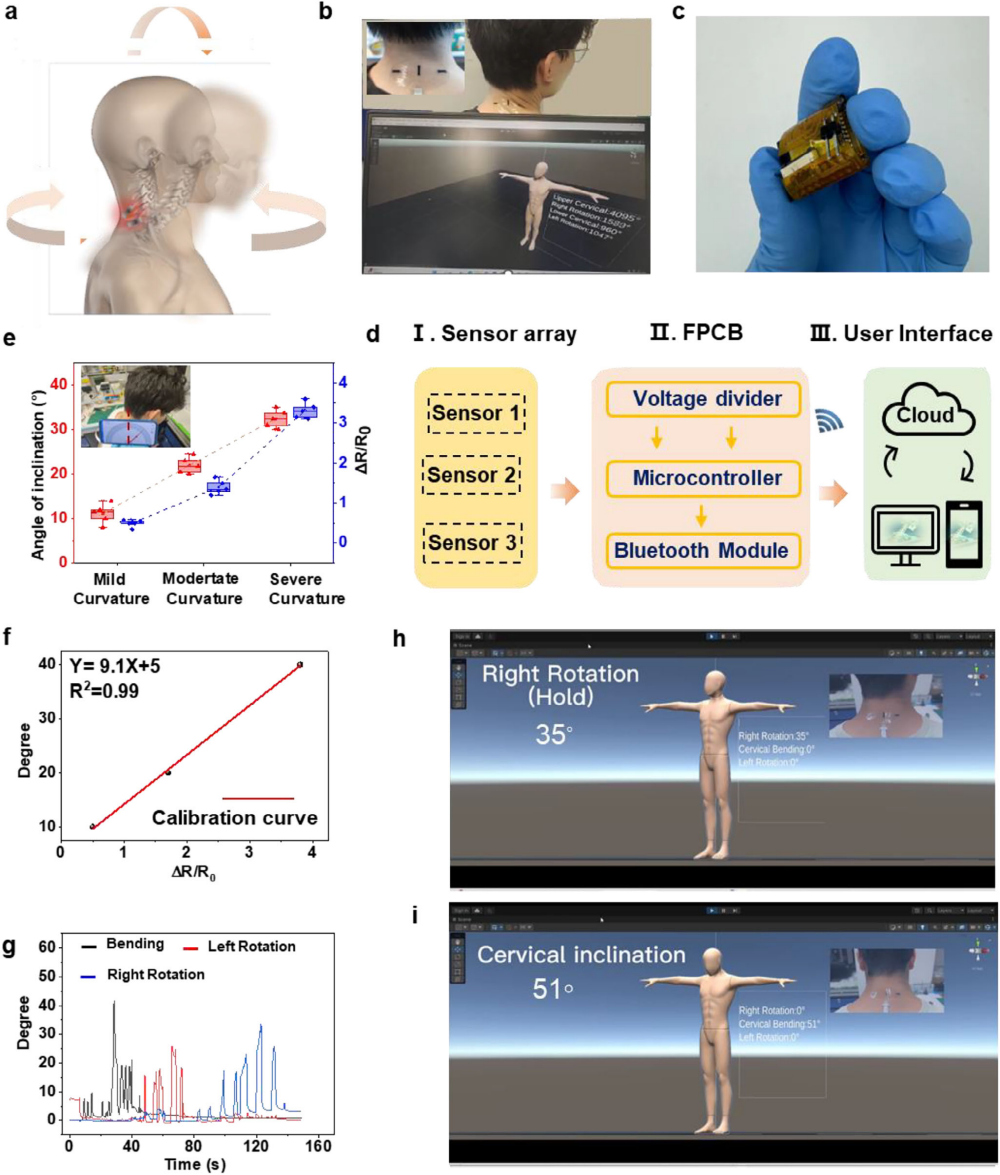
a) Schematic diagram of three typical movements of the human cervical spine, including twisting to the left, twisting to the right, and bending forward. b) Optical images of the sensor array attached to the cervical spine for monitoring cervical spine conditions. c) PCB of the wireless monitoring system for communicating with a laptop and signal processing. d) Block diagrams of the system. e) Resistance changes and the calculated angles from the sensors when the user bends their cervical spine at different amplitudes. f) Calibration curve for calculating the bending angles. g) Calculated angles based on the calibration curve and output signals of the sensor array during three kinds of cervical spine motions. A custom‐designed Unity‐based avatar for monitoring cervical spine h) rotation and i) bending.

The degree of cervical spine motion is closely correlated with musculoskeletal health and can serve as a quantitative biomarker for various cervical spine conditions.^[^ ^7^ ^]^ Specifically, a reduced range of motion (ROM) in flexion, extension, lateral bending, or rotation may suggest underlying issues such as cervical spondylosis, disc herniation, or muscle stiffness, often resulting from pain, inflammation, or mechanical restriction.^[^ ^8^ ^]^ Asymmetrical cervical spine movements between the left and right sides can indicate muscle imbalances, neurological disorders, or postural compensation, potentially leading to chronic discomfort or injury if left unaddressed. Prolonged poor posture, such as sustained forward flexion exceeding 40°, is strongly associated with text cervical spine syndrome and contributes to chronic pain, muscular fatigue, and spinal degeneration.^[^ ^9^ ^]^ Because of the high capture accuracies of the joint motion from iStretch, we developed a monitoring system to visualize cervical spine motion for the potential of early detection of dysfunctions, rehabilitation monitoring, proactive behaviour modification, and posture correction, offering significant benefits for long‐term spinal health management. As depicted in **Figure** **6**a, this application investigates three typical movements of the human cervical spine: rotation to the right, rotation to the left, and bending. To effectively monitor these movements and reduce crosstalk between signals, the monitoring platform is designed with three iStretch sensors in three directions. Given that cervical movement is based on a complex mechanism involving eight joints and muscle cooperation (i.e., the skull and seven cervical vertebrae C1–C7),^[^ ^70^ ^]^ it is sensible to position the iStretch sensor array close to the cervical vertebrae and related muscles. Thus, we adhere one sensor to the skin surface of the cervical spine joints, aligned with the vertical direction of the cervical spine to monitor bending status, and the other two along the horizontal line of the two side cervical spine muscles to monitor cervical spine rotation, respectively (Figure 5b). Due to their high flexibility and softness, the sensor array can easily conform to the skin surface of the cervical spine during movement without any discomfort or detachment.

To facilitate long‐term and real‐time monitoring of the cervical spine motions, the iStretch sensory platform was integrated with a wireless miniaturized flexible programmable system on a chip (PSoC), as depicted in Figure 6c. Figure 6d illustrates the block diagram of the system's internal components, which consist of three main sections: the microcontroller, voltage divider, and Bluetooth module for measuring sensor outputs and wireless transmission. Generally, cervical spine movements cause the extension of the sensor array, leading to corresponding variations in output electrical signals due to the direct piezoresistance effect. These electrical signals are continuously transmitted via Bluetooth to intelligent terminals (e.g., phones, computers) for further data storage and analysis.

To validate the feasibility, we qualitatively analyzed the three motion modes by our sensor array, simultaneously recorded the angle change of the cervical spine by placing an angulometer right in the middle position, and then obtained the calibration curve for angle prediction. Taking the bending motion as an example, when a subject made a forward flexion with mild/moderate/severe amplitudes, the angulometer gave outputs of 10°, 20°, and 40°, respectively, and our device delivered average resistance changes of about 0.5, 1.7, and 3.8 (Figure 6e). By correlating the bending angles and electrical signal variation, we can get the calibration curve for calculating the bending angles (Figure 6f). The corresponding curves for the rotation modes can be obtained in the same way as well (Figure S22, Supporting Information).

Based on the calibration curves and the resistance changes recorded from our sensor array, real‐time continuous bending and rotation angles can be calculated to monitor the cervical spine conditions and digitally mimic the clinical assessment of cervical spine movements, as shown in Figure 6g. As a proof of concept, we developed a graphical user‐interactive interface based on Unity Engine. Through this interface, the quantitative bending, right rotation, and left rotation angles and corresponding cervical spine conditions can be shown to the user in real‐time (Figure 6h,i; Figure S23, Supporting Information). Thus, this interface can serve as a monitoring system for self‐assessment and help patients who have undergone cervical spine surgery to evaluate the recovery process or help people with chronic cervical spine diseases assess the severity of their conditions (Video S4, Supporting Information). Instead of using bulky medical imaging instruments or relying on subjective visual assessment by clinical specialists observing patients’ cervical spine movements, hospitals with outpatients can use the device to quantitatively assess the patients’ cervical spine conditions as well. By allowing users to record their cervical spine motion in the comfort of their homes, it mitigates the necessity for frequent hospital visits, thereby potentially expediting the recovery process. Furthermore, the collected raw data can be seamlessly transmitted to cloud servers. This data accessibility empowers healthcare specialists to remotely assess patients' health conditions with precision and timeliness. Consequently, this technology exhibits immense potential in modern medical Internet of Things (IoT) systems, providing high degrees of accessibility, immediacy, and accuracy. Our method allows for the visualization of the assessment process through a customized avatar, which enables the tracking of the user's motion and simultaneously analyses the collected data.

## 3 Conclusion

We presented a user‐interactive strain‐sensitive iStretch for omnidirectional biomechanical detection, visualized strain sensing, and thermotherapy, joint and spine health management. The iStretch consists of a strain‐sensitive m‐GLM composite layer and a thermochromic layer. Due to the interplay of bulk liquid metal, liquid metal particles, and graphene network, the m‐GLM layer mitigates issues of cracking and delamination and contributes to high strain‐resilience and broad working ranges. This overcomes the traditional limitation of the trade‐off between high gauge factor and a broad sensing range, thus enhancing the overall strain‐sensing performance. The thermochromic layer allows the iStretch device to visually display strain through color change and alert users to overheating during thermotherapy. We demonstrated that the iStretch can detect omnidirectional motions, from large‐strain gestures such as arm bending to small‐strain signals such as a pulse and breathing. Additionally, an intelligent motion identification CNN model was developed to analyse and classify user activities for behavioural studies. We also demonstrate that the device can act as a user‐interactive thermotherapeutic tool, allowing users to adjust its surface temperature according to the color change while synchronizing with body movements. This feature can help treat tendon, ligament, and joint injuries while providing visual cues to prevent overheating. Finally, we fabricate a sensor array for quantitative cervical spine assessment. Overall, our user‐interactive iStretch offers a promising approach to monitor joint chronic illnesses, provide thermotherapy, and build a comprehensive closed‐loop health‐monitoring and treatment system.

## 4 Experimental Section

### Raw Materials

EGaIn (Ga, 75.5%, and In, 24.5% by weight; melting point, ≈15.7 °C) and cyclohexanone solvent were purchased from Sigma–Aldrich. Microencapsulated thermochromic dyes (green, red, and blue) were purchased from Dongfang Color Change Technology Co., Ltd. (Shenzhen, China). Graphene powder was purchased from Huiheng Graphite Technology Co., Ltd. (Qingdao, China). Polyester polyol‐rich thermoplastic polyurethane (pp‐TPU) particle (Pearlstick 5703) was purchased from Lubrizol Co., Ltd. All the above reagents were used without further purification.

### Preparation of EGaIn/Graphene Ink

First, 10 mg mL^−1^ graphene dispersion was made by dispersing graphene powder in cyclohexanone solution, followed by ultrasonication for 120 min using a probe sonicator (Sonics VC750, 750 Watts, set at 100% amplitude). After that, a certain amount of EGaIn was added to the uniform graphene solution, followed by 2 min of ultrasonication. The composite solution was then transferred to a centrifuge (Mikro 220, set at 5000 RPM) to obtain the EGaIn/Graphene composite slurry. Finally, TPU/cyclohexanone solution (weight ratio 1:5) and the above slurry were mixed for 30 min at 2000 rpm using a planetary mixer (Speedmixer DAC330‐100 SE) to form a homogeneous printable EGaIn/Graphene composite ink.

### Preparation of the Thermochromic Elastomer

The dynamic thermochromic dyes were made by mixing the red, green, and blue dyes in a weight ratio of 1:4:5. In order to improve the printability and uniformity of dynamic thermochromic dyes, TPU/cyclohexanone solution of equal weight was added into the mixture, which was then magnetically stirred for 1 h at room temperature to obtain a uniform printable thermochromic composite.

### Fabrication of Composite‐Based Strain Sensor

Screen‐printing method was used to fabricate a specific pattern device. First, a commercial paper‐cutter machine was applied to cut the double‐adhesive PI tape into different patterns as masks. Next, the craved double tap was covered on the substrate. Then, printing EGaIn/Graphene ink was then stencil printed through a roller on substrates, and the masks were peeled off from the substrates. The printed patterns were finally cured in the oven at 70 °C for 30 min, after that, the dynamic thermochromic dyes were cast onto the work as a visualization layer as well as a packaging layer. After that, a cyclic stretching activation step (applied strain: 100%,100 cycles) was implemented to intentionally induce controlled cracking in the graphene‐liquid metal (LM) composite for pre‐stabilizing the initial resistance.

### Interconnects between Printing Composite Pads for External Wiring

A hybrid interconnection strategy using anisotropic conductive film (ACF, 3 M 7303) was implemented to bridge the m‐GLM composite and conventional metal contact pads. The ACF offers both mechanical adhesion and vertical electrical conductivity, while its anisotropic properties minimize lateral shorting, thereby preserving signal integrity under strain. Additionally, a thin layer of extruded liquid metal was printed onto the contact pads prior to bonding to enhance the conductive interface and improve electrical connectivity. Before lamination, the surfaces of both the m‐GL composite and the metal pads underwent oxygen plasma treatment to improve surface energy and promote strong interfacial adhesion. The bonded assembly was then subjected to compression under controlled pressure to ensure consistent bonding across the contact area. This interconnect configuration was found to be mechanically stable and electrically reliable during repeated deformation, making it well‐suited for long‐term operation in a stretchable bioelectronic system.

### Sample Characterization

The strain‐resistance tests were carried out on a high‐precision electronic universal testing machine (Shimadzu testing platform) with a Keithley 6400 digital multimeter used to monitor the resistance change of testing samples with a length of 30 mm and a width of 10 mm. The SEM characterizations were carried out using field‐emission scanning electron microscopy (JSM‐7800) at an accelerating voltage of 20 kV. The temperature of the device surface was measured using an IR camera (E80, FLIR Systems, Inc.), and the FLIR tool was used for analysis.

### Statistical Analysis

Electromechanical traces were baseline‐corrected and expressed as relative resistance change (ΔR/R₀). Data in line charts are displayed as mean ± SD. All statistical analyses were performed using OriginPro 2018 (OriginLab, USA) for mechanical and thermal datasets, and Python 3.11 with scikit‐learn 1.5 for CNN model training and confusion matrix evaluation.

### Human Subject Study

The human subject study was conducted with the participation of 10 healthy volunteers, adhering to the IRB‐approved protocol (NUS‐IRB‐2023‐828) from the National University of Singapore Institutional Review Board. The data of motion tracking collected in the human subject study were approved by the volunteers, who signed consent forms prior to the experiments.

## Conflict of Interest

The authors declare no conflict of interest.

## Author Contributions

C.T.L., S.W.C., and S.C.F. conceived the idea. S.C.F. and S.W.C. designed the experiments and contributed equally to this work. S.C.F. completed most of the experiments. J.M.Q. designed the Unity software for displaying cervical spine monitoring applications. S.C.F. and S.W.C. drafted the manuscript. J.M.Q., Z.Q., Z.X.W., and C.T.L. contributed to discussing and finalizing the manuscript.

## Supporting information



Supplemental Video 1

Supplemental Video 2

Supplemental Video 3

Supplemental Video 4

Supporting Information

## Data Availability

The data that support the findings of this study are available from the corresponding author upon reasonable request.
