# Supplementary material for: Strain‐Sensitive Thermochromic Smart Electronic Skin for Joint and Spine Healthcare Applications
Source: Adv Sci (Weinh). 2025 Jul 6;12(37):e07605. doi: 10.1002/advs.202507605 (PMC12499495; doi:10.1002/advs.202507605)
Supplement: Supplementary file 5 — Supporting Information [file ADVS-12-e07605-s005.docx]

Supporting Information

**STRAIN-SENSITIVE THERMOCHROMIC SMART E-SKIN FOR JOINT AND SPINE HEALTHCARE APPLICATIONS**

Shicheng Fan, Shuwen Chen*, Zheng Qiao, Jiaming Qi, Zixiong Wu and Chwee Teck Lim*

S. C. Fan, Z. Qiao, J. M. Qi, Z. X. Wu, Prof. C. T. Lim

Department of Biomedical Engineering

National University of Singapore

Engineering Drive 3, Singapore 117583, Singapore

E-mail: [ctlim@nus.edu.sg](mailto:ctlim@nus.edu.sg)

Dr. S. W. Chen,

Institute of Medical Equipment Science and Engineering

Huazhong University of Science and Technology

Luoyu Road 1037, Wuhan 430074, China

E-mail: [shuwenchen@hust.edu.cn](mailto:shuwenchen@hust.edu.cn)

Prof. C. T. Lim

Institute for Health Innovation and Technology (iHealthtech)

National University of Singapore

15 Kent Ridge Crescent, Singapore 119276, Singapore

Prof. C. T. Lim

Mechanobiology Institute

National University of Singapore

Engineering Drive 1, Singapore 117411, Singapore

Table of contents

Supplementary Figures

[Figure S1. Characterization of LM droplets. 4](#_Toc198253284)

[Figure S2. Fabrication process for M-GLM and thermochronic dye-based iStretch 4](#_Toc198253285)

[Figure S3. Customization of iStretch 5](#_Toc198253286)

[Figure S4. Machine controlled scalable printing 5](#_Toc198253287)

[Figure S5. High-resolution printing over different substrates. 6](#_Toc198253288)

[Figure S6. Contact angle comparison of eGaIn and M-GLM ink on various substrates. 6](#_Toc198253289)

[Figure S7. Dynamic cycling resistance change under different strains. 7](#_Toc198253290)

[Figure S8. Stretchability of M-GLM. 7](#_Toc198253291)

[Figure S9. Gauge factors for iStretch strain sensor. 8](#_Toc198253292)

[Figure S10. Response time 8](#_Toc198253293)

[Figure S11. Optical images of iStretch under different strains 9](#_Toc198253294)

[Figure S12. SEM images show the interplay of EGaIn and graphene 9](#_Toc198253295)

[Figure S13. Simulated stress-displacement comparison of device models with only graphene and M-GLM composite. 10](#_Toc198253296)

[Figure S14. The resistance change of the iStretch during wrist bending 10](#_Toc198253297)

[Figure S15. Resistance change of the iStretch during small biomechanical activities. 11](#_Toc198253298)

[Figure S16. Raw data of different body motions for training CNN model 11](#_Toc198253299)

[Figure S17. Color change mechanism of iStretch under different temperatures. 12](#_Toc198253300)

[Figure S18. Temperature and color change of the iStretch with the increasing applied current. 12](#_Toc198253301)

[Figure S19. Heating alert mechanism 12](#_Toc198253302)

[Figure S20. Temperature coefficient of resistance of m-GLM 13](#_Toc198253303)

[Figure S21. Comparison of the coefficient of variation between pre- and post-thermotherapy. Black dot represents pre-thermotherapy, and Red dot represents post-thermotherapy. 13](#_Toc198253304)

[Figure S22. Qualitatively analysing motion modes of the neck 14](#_Toc198253305)

[Figure S23. A custom-designed Unity based avatar for monitoring left neck rotation. 14](#_Toc198253306)

[Supplementary Video 1. Self-healed iStretch sensor under tensile condition. 14](#_Toc198253307)

[Supplementary Video 2. Color change of thermochromic composites with temperature increase. 14](#_Toc198253308)

[Supplementary Video 3. Strain dependent thermochromic changes in iStretch during various thermotherapy applications. 14](#_Toc198253309)

[Supplementary Video 4. A custom-designed Unity based avatar for neck motion monitoring. 14](#_Toc198253310)

[Table S1. Performance comparison between literature and our work 15](#_Toc198253311)


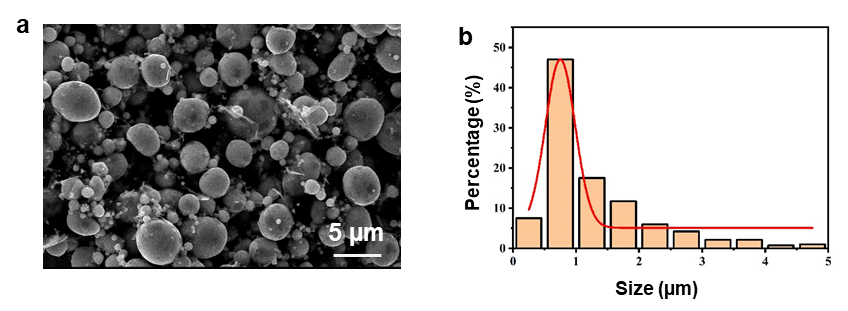


# **Figure S1.** Characterization of LM droplets.

a) SEM image and b) diameter distribution of LM droplets. The LM particle dispersion was fabricated by ultra-sonicating LM for 2 min. The size distribution of the LM droplets ranges from 500nm to 5 µm.


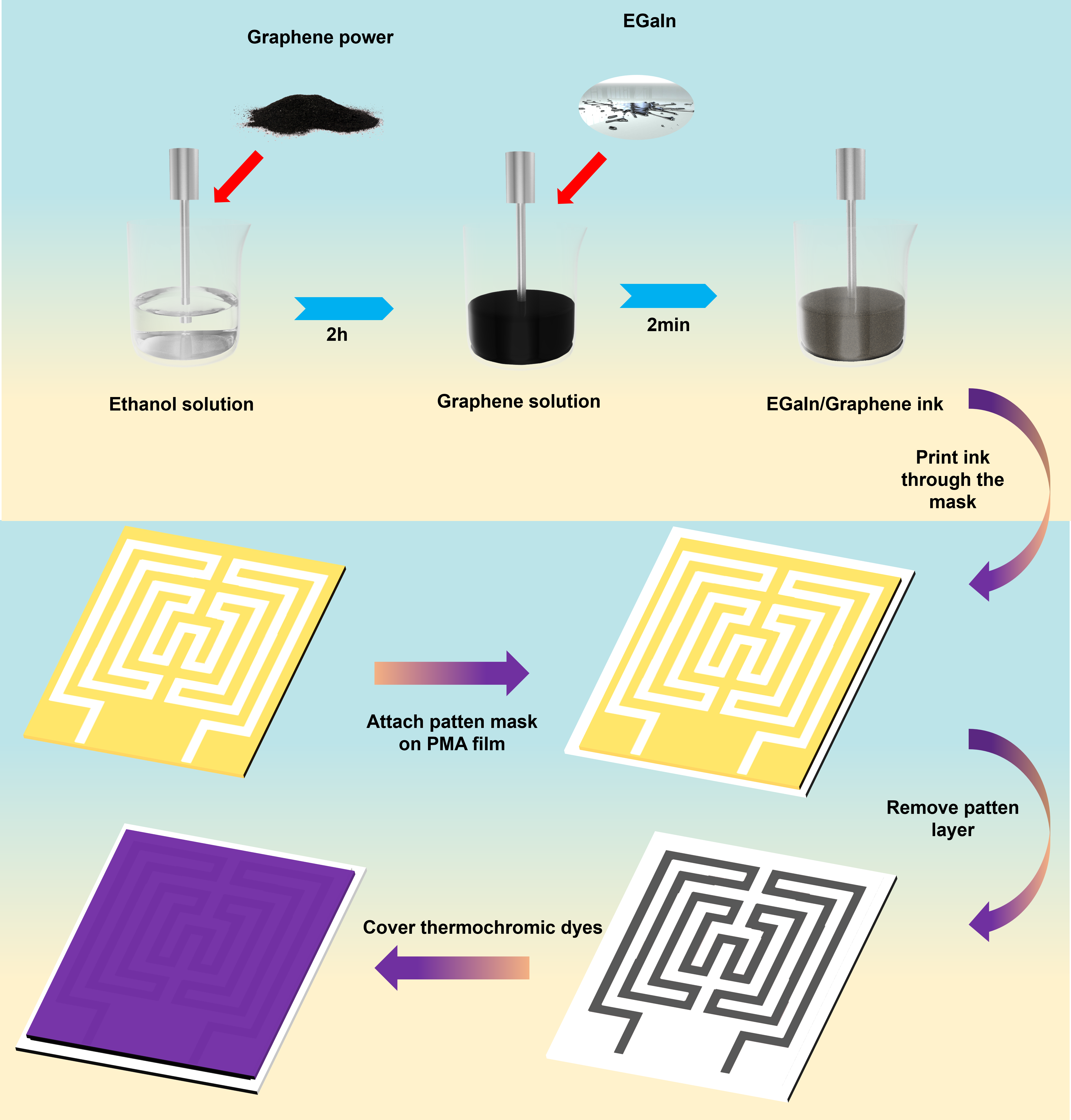


# Figure S2. Fabrication process for M-GLM and thermochronic dye-based iStretch

Illustration of facile fabrication process for EGaIn/Graphene composite.

**
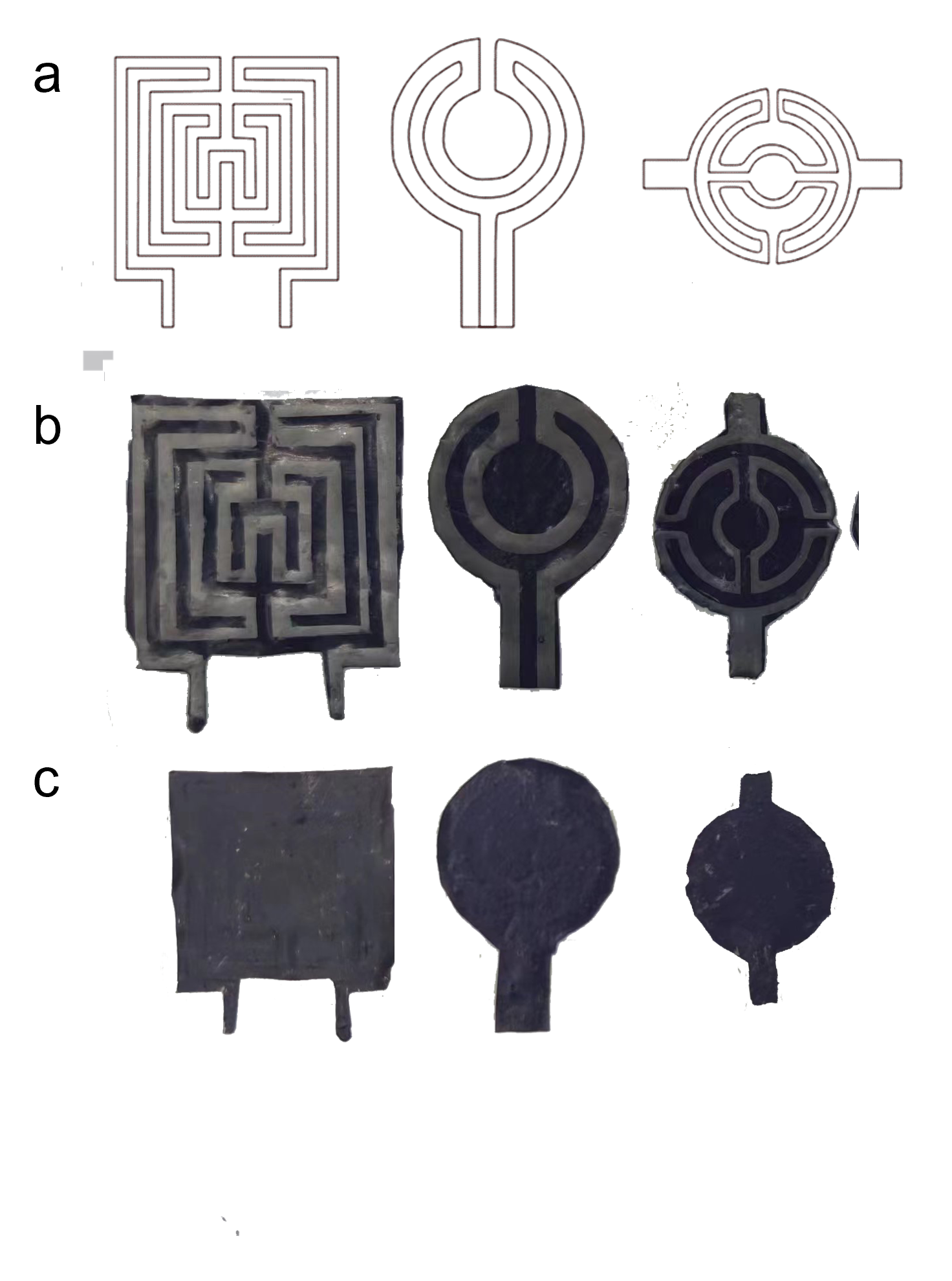
**

# Figure S3. Customization of iStretch

Customized shape and size for different body areas. a) The mask image for different patterns. b,c) the front and back side of iStretch patch.

**
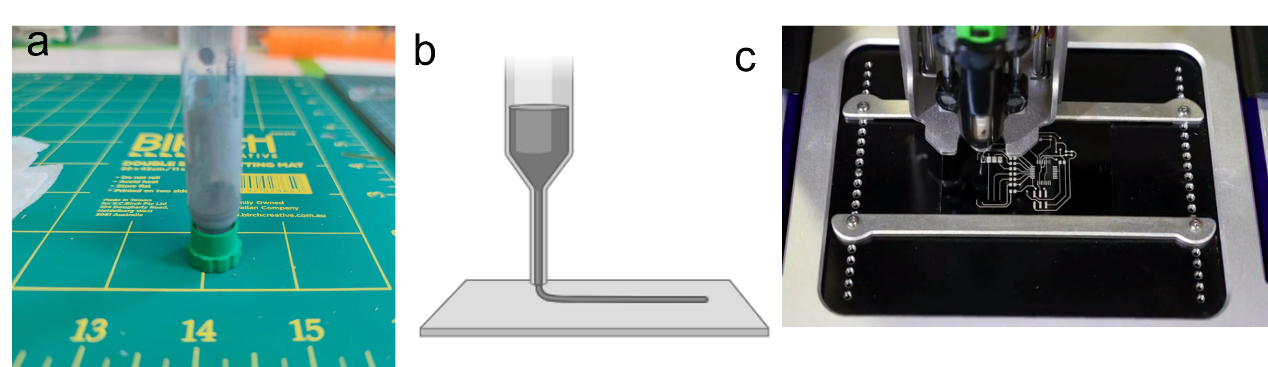
**

# Figure S4. Machine controlled scalable printing

a) M-GLM composite ink in the cartridges. b) Illustration of the ink extruded from the printer nozzle. c) Illustration of low-cost desktop extrusion printer (Voltera) to print various patterns.


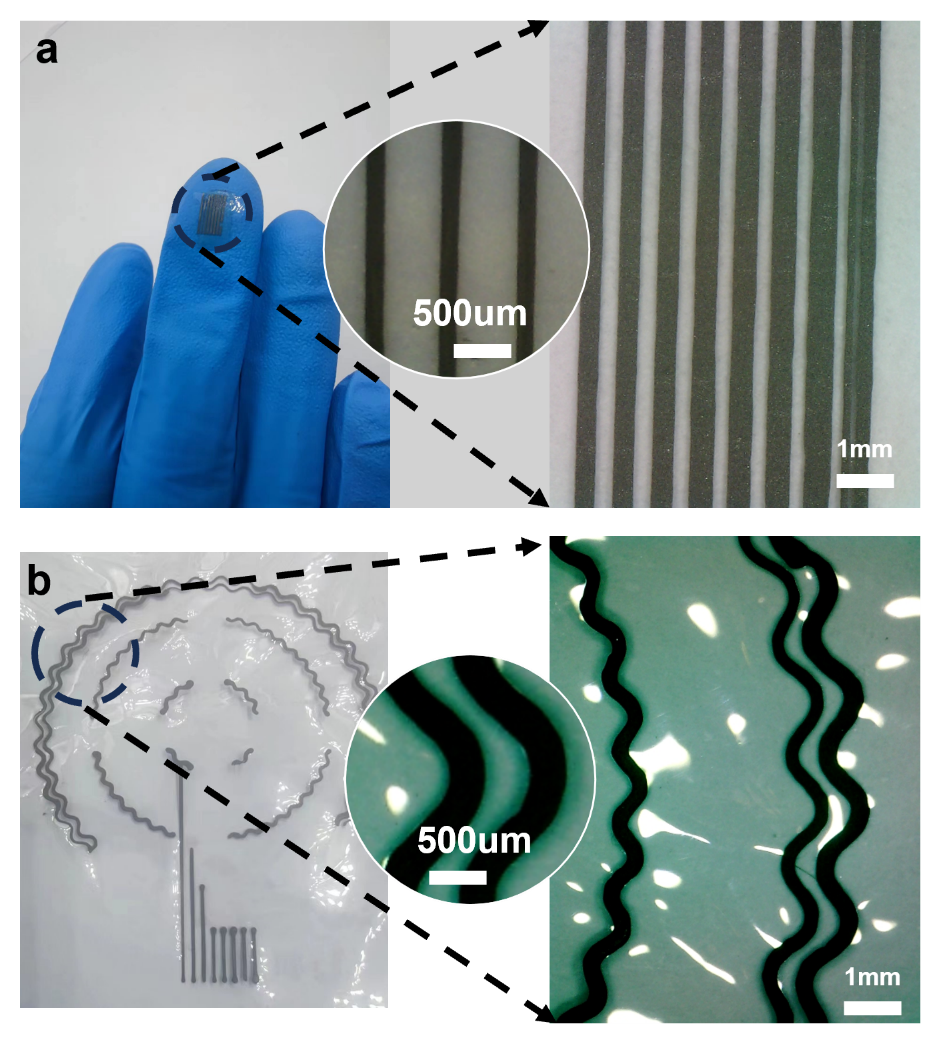


# Figure S5. High-resolution printing over different substrates.

Straight trace on SEBS substrate. b) Serpentine trace on TPU substrate.

**
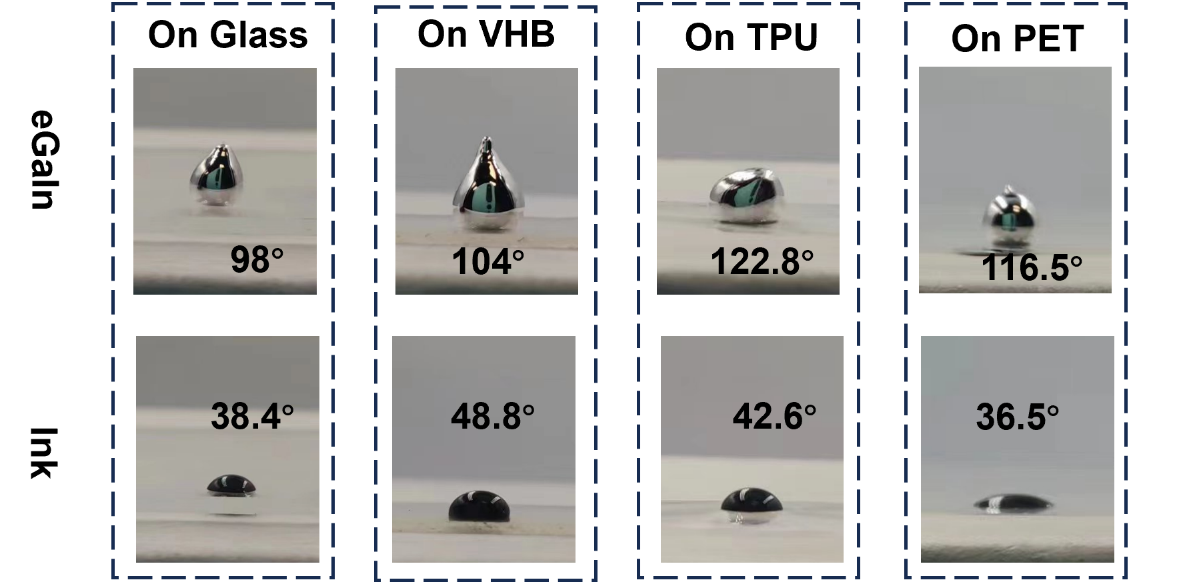
**

# Figure S6. Contact angle comparison of eGaIn and M-GLM ink on various substrates.

The contact angle of eGaIn and M-GLM ink on a) glass, b) VHB, c) TPU and d) PET substrates, respectively.


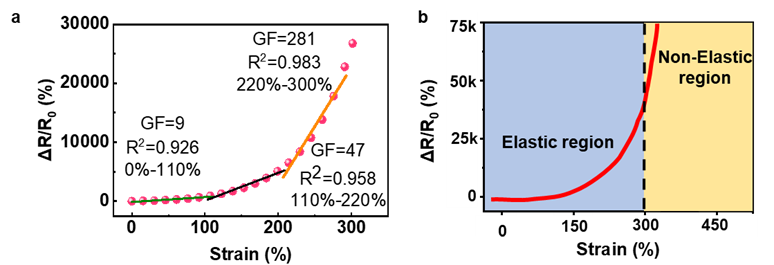


# Figure S7. Gauge factors for iStretch strain sensor.

(a) For strains ranging from 0 to 110%, the GF is relatively low at 9.3. This can be attributed to the anchoring effect of the EGaIn inside the graphene layers, which restricts the slipping of the graphene layers during this initial strain range. As the strain increases beyond 110%, the GF rises significantly, reaching 47, and eventually peaks at 281 for elongations up to 300%. This increase in GF at higher strains suggests a progressive decoupling of the graphene layers, allowing for more pronounced deformation and enhanced sensitivity. (b) The dashed line indicates the critical strain (εc=300%) separating the elastic (reversible) and non-elastic (irreversible) regions. Beyond εc, the resistance increase becomes permanent due to microstructural damage and the cyclic strain tests show irreversible resistance change.


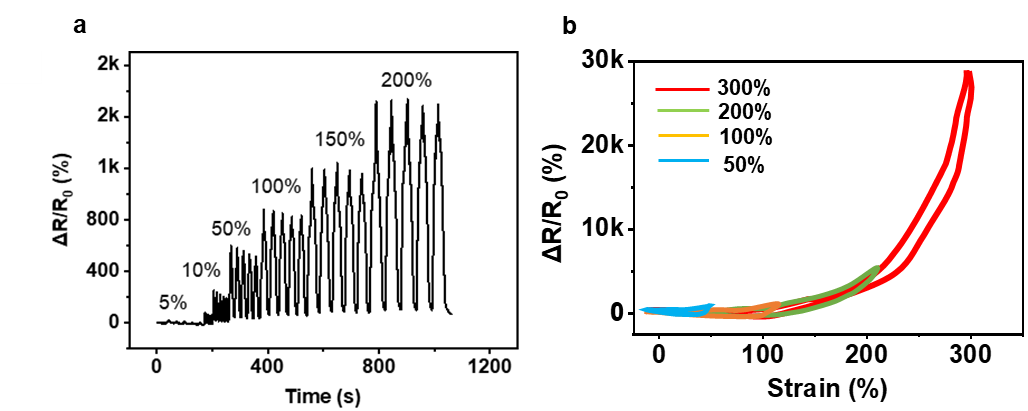


# Figure S8. Dynamic cycling resistance change under different strains. (a) Cyclical stretching/release test under a gradient strain range from 1% to 150%. (b) To evaluate the hysteresis effect, loading–unloading cycles were conducted, and the resistance response exhibited negligible hysteresis over a wide strain range up to 200%. Specifically, the hysteresis values were measured as 5.73% ± 0.33% at 50% strain, 6.95% ± 0.14% at 100% strain, and 7.73% ± 0.40% at 200% strain, 10.44% ± 0.71% at 300% indicating excellent mechanical reversibility. These results demonstrate outstanding performance—particularly under large strain condition—highlighting the sensor’s robustness and suitability for dynamic wearable applications.


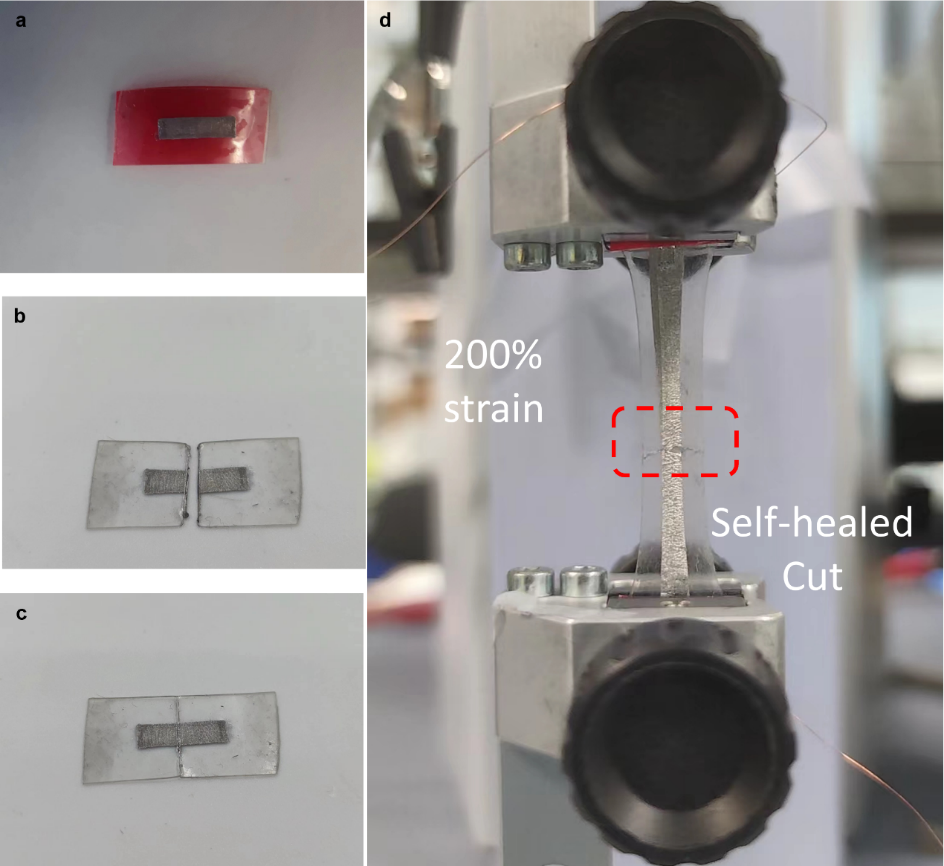


# Figure S9. Stretchability of M-GLM.

Photographs of M-GLM sensor after cutting and healing stretching under a strain of ≈200%.


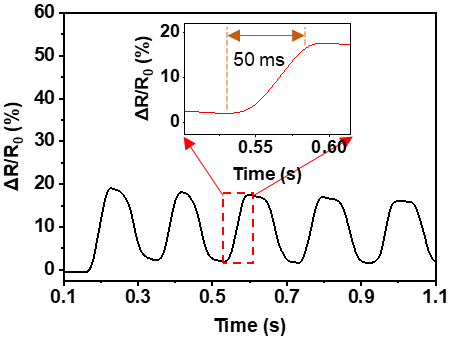


# Figure S10. Response time.

Fast response time. Inset: Zoom-in of the selected area. To accurately determine its responding speed, a 1% strain with a speed of 10 mm s^−1^ was applied to the sensor.


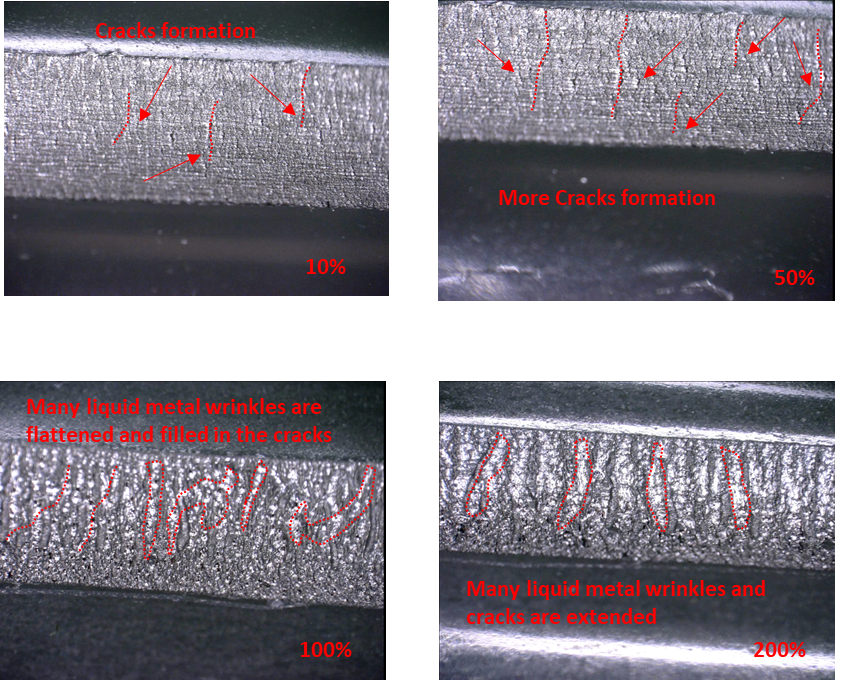


# Figure S11. Optical images of iStretch under different strains.

The liquid metal bridges the crack and stretches with strain, ensuring high strain sensing range and resilience.


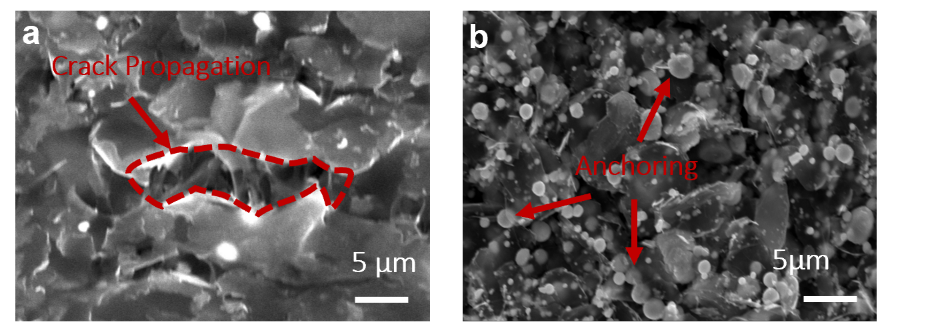


# Figure S12. SEM images show the interplay of EGaIn and graphene.

SEM images show EGaIn particles sandwiched by graphene nanosheets and sinter EGaIn connected adjacent graphene nanosheets


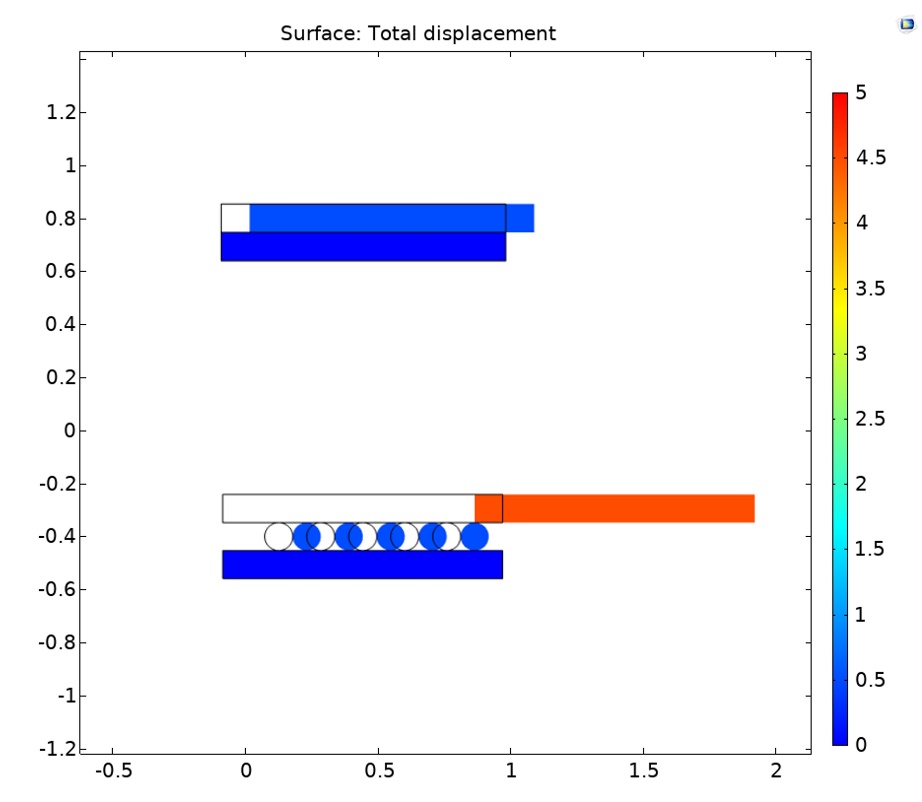


# Figure S13. Simulated stress-displacement comparison of device models with only graphene and M-GLM composite

The displacement differences between pure graphene network (upper) and EGaIn decorated graphene network (bottom) structures under same pulling force as analyzed by finite element simulation (FES).


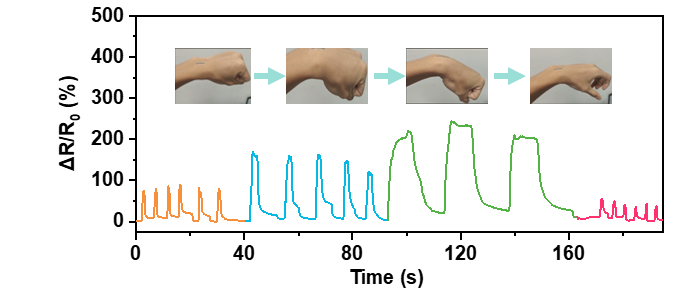


# Figure S14. The resistance change of the iStretch during wrist bending

The wrist movement could be detected and with motion amplitude increase, the output signal will change accordingly.


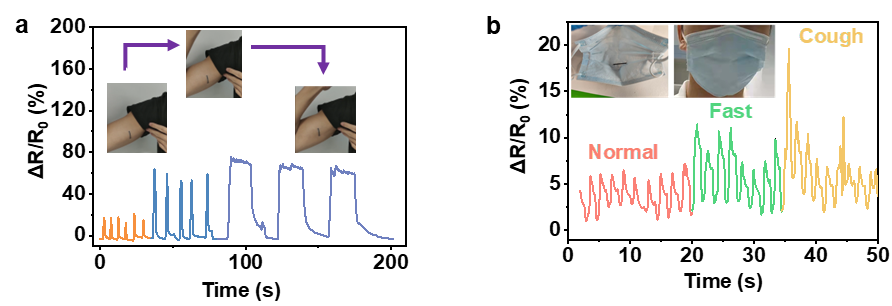


# Figure S15. Resistance change of the iStretch during small biomechanical activities

1. Resistance change of iStretch during muscle flexing activities. b) Detecting different respiration modes. Insets are the digital photographs of the devices printed on a fabric mask and worn by a tester.


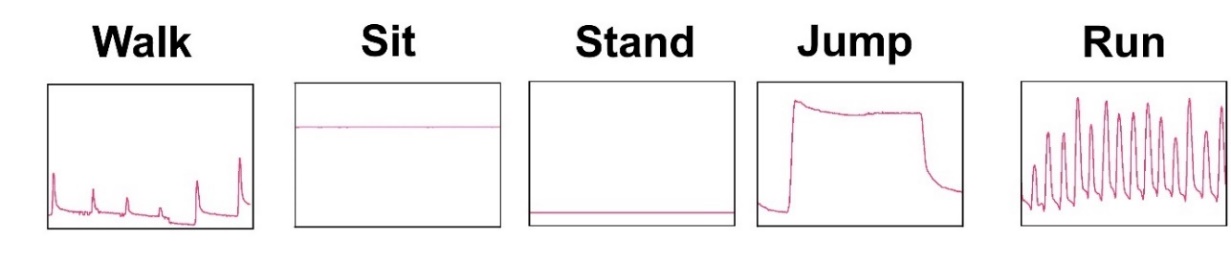


# Figure S16. Raw data of different body motions for training CNN model


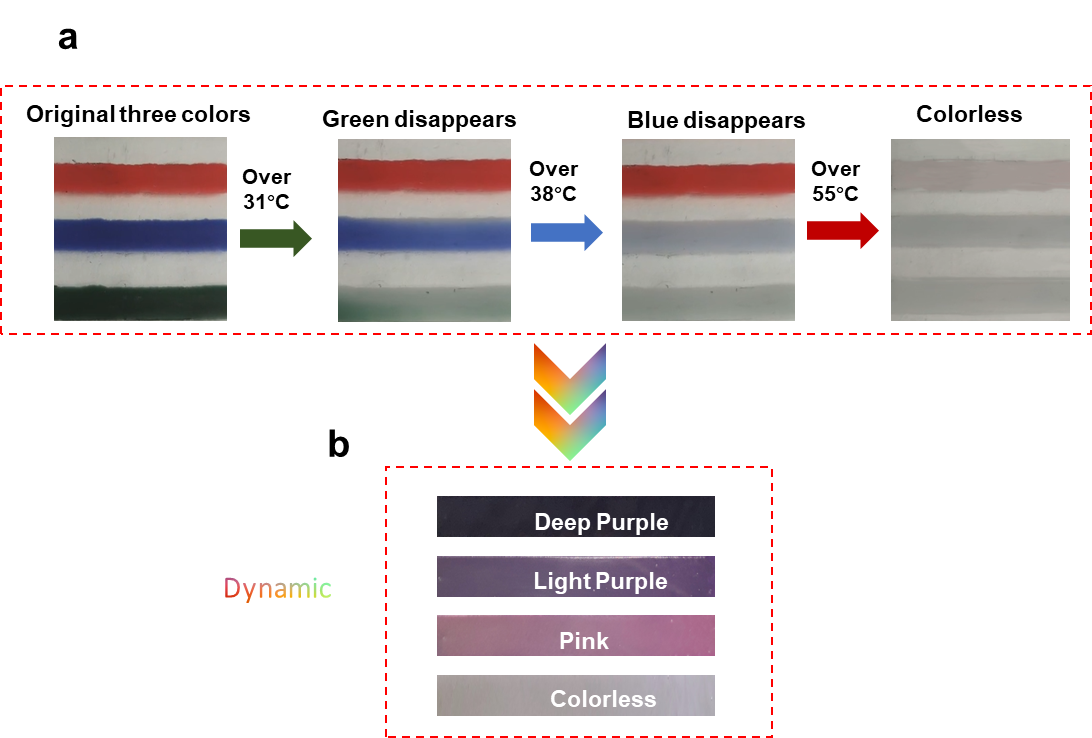


# Figure S17. Color change mechanism of iStretch under different temperatures

1. Three primary color units (red, blue, green) were used to prepare the dynamic dyes for optimizing the discrimination effect of color changing. b) Composite layer color transitions process.


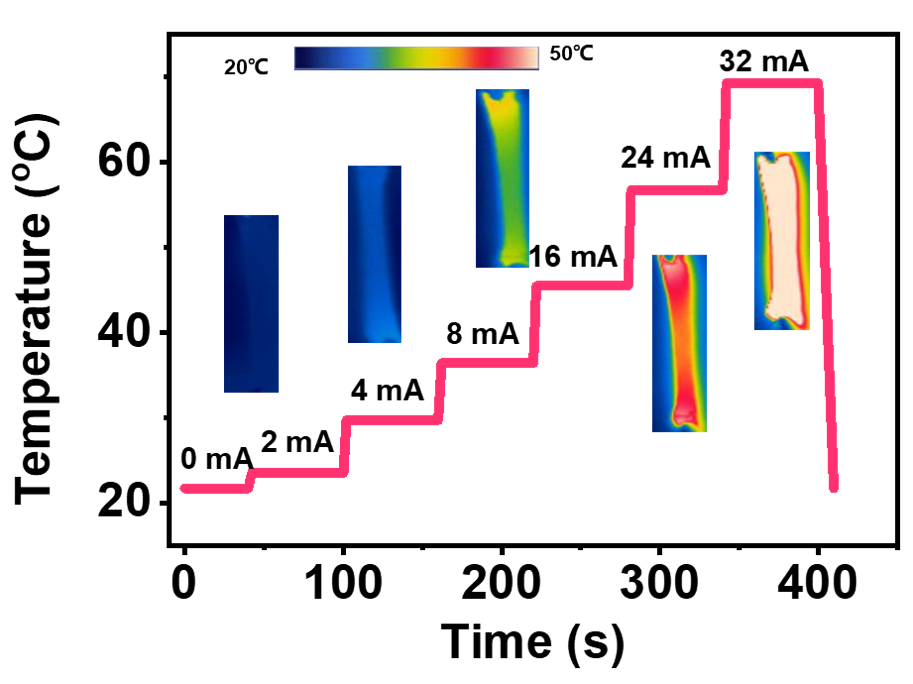


# Figure S18. Temperature and color change of the iStretch with the increasing applied current

Time-dependent temperature change as a function of applied current from 0 to 20 mA.


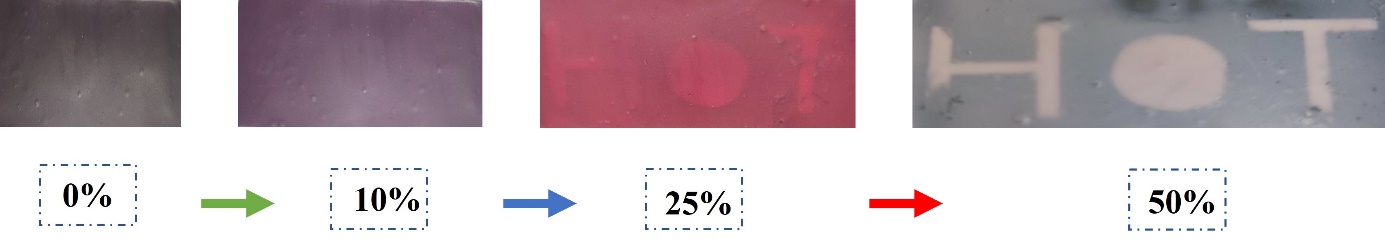


# Figure S19. Heating alert mechanism

Our device could be applied to visualize strain alert for indicating and preventing critical strain limits.

# Figure S20. Temperature coefficient of resistance of M-GLM.

The variation of resistivity with temperature is linear and relatively minor within moderate temperature ranges.

# Figure S21. Comparison of the coefficient of variation between pre- and post-thermotherapy. Black dot represents pre-thermotherapy, and Red dot represents post-thermotherapy.

**
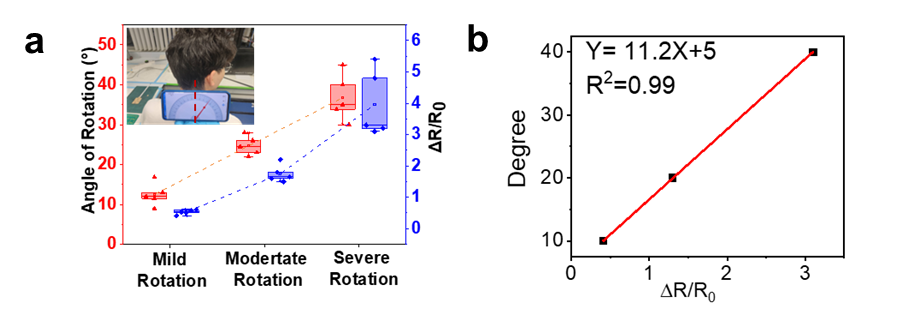
**

# Figure S22. Qualitatively analysing motion modes of the neck.

1. Resistance changes of iStretch under neck rotation. b) Correlation between rotation angle and electrical signal variation.


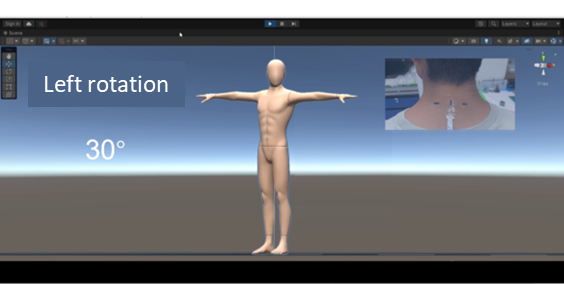


# Figure S23. A custom-designed Unity based avatar for monitoring left neck rotation.

**Supplementary Movies**

# Supplementary Video 1. Self-healed iStretch sensor under tensile condition.

# Supplementary Video 2. Color change of thermochromic composites with temperature increase.

# Supplementary Video 3. Strain dependent thermochromic changes in iStretch during various thermotherapy applications.

# Supplementary Video 4. A custom-designed Unity based avatar for neck motion monitoring.

# Table S1. Performance comparison between literature and our work

|  | **Materials** | **Sensing mechanism** | **ε_max_ (%)** | **ΔR(t)/R_0_ at max strain** | **Functions** | **Ref** |
| --- | --- | --- | --- | --- | --- | --- |
| 1 | EGaIn tube | Shape deformation | 180% | 6 | Physiological measurements from different body parts. | ^[1]^ |
| 2 | Graphene film | Appearance and propagation of cracks | 35% | 502 | Monitoring human vital signals and in appliances with increased human–machine interaction. | ^[2]^ |
| 3 | EGaIn coated fiber | Shape deformation | 300% | 2 | High strength conductor, heater, and stretchable wire | ^[3]^ |
| 4 | Multiwall carbon nanotube (MWCNT) film | Appearance and propagation of cracks cooperated with chiral auxetic metamaterial | 30% | 25542 | On-body monitoring of human physiological signals and a smart training assistant for trampoline gymnastics | ^[4]^ |
| 5 | Mazelike vertical graphene film | Microcracks are initiated in the buffer layer | 120% | 88.4 | Wearable sensors for human motion detection and pressure distribution measurement | ^[5]^ |
| 6 | 3D graphene foam/CNTs | Deformation by producing cracks inside the 3D microarchitectural graphene foams | 85% | 35 | Wearable electronic device for detecting of electrophysiological stimuli and even for acoustic vibration recognition. | ^[6]^ |
| 7 | LM@rGO@PAA composite (PAA, poly(acrylic acid)) | A deformable ionic conductor connects cracked graphene sheets electronically, enabling the strain sensor to be stretched to 600% of its original length | 600% | 9.86 | Multiple sensations such as high sensitivity to pressure /strain, temperature evolution, response to solvent change, and sensing atmospheric negative pressure (vacuum). | ^[7]^ |
| 8 | LM-silicone (LMS) inks | Shape deformation | 100% | 1.1877 | LM-based flexible electronics, multilayer soft circuits, strain sensors, and data gloves | ^[8]^ |
| 9 | liquid metal (LM)/ acrylic acid (AA) | Shape deformation from three-dimensional (3D) hydrogel networks | 500% | 1.54 | flexible strain-sensing devices | ^[9]^ |
| 10 | Graphene/CNT /BCN/TPU fiber | The porous structure in TCG-BCN fiber enables the strain sensor to uniformly withstand external forces | 130% | 1501 | Wearable fiber-based strain sensing | ^[10]^ |
| 11 | Polymer Nanoball Decorated graphene PU fiber | The designed graphene frames the porous graphene fibers undergoes larger structural variations and more conducting-network changes | 8% | 87 | Fiber-based textile sensors to recognize a pulse wave and eyeball movement in real-time and distinguish the multilocation in real time | ^[11]^ |
| 12 | Graphene /(PEDOT:PSS) | Synergistic enhancement in electrical conductivity by π-π interaction and charge delocalization between graphene and  PEDOT:PSS. | 40% | 500 | Monitor electrophysiological signals such as facial skin and brain activity with low-motion artifact | ^[12]^ |
| 13 | Substrate-free laser scribed graphene (SFG) | Propagation of cracks | 100% | 316 | ECG detecting system | ^[13]^ |
| 14 | LM/RGO@C/Rubber composite (LM, liquid; RGO; C, cotton burning-based carbon) | Propagation of cracks | 515 % | 1.63 × 10^5^ | Monitoring of full-range (vigorous and subtle) human motions and physiological activities | ^[14]^ |
| 15 | Hybridized metal–organic framework (MOF)-derived porous carbon (HPC) deposited on laser-engraved graphene (LEG) | Propagation of cracks | 20% | 575,542 | Monitoring of full-range (vigorous and subtle) human motions and physiological activities | ^[15]^ |
| 16 | Thermoplastic polyurethane/graphene | Propagation of cracks | 150% | 10.551 | Strain sensor for structural health monitoring and wearable devices | ^[16]^ |
| 17 | Waterproof graphene (GR)/carbon nanotube  (CNT)/Polydimethylsiloxane (PDMS) composites. | The graphene plates are dispersed  uniformly in the PDMS matrix, and carbon nanotubes act as  bridges to connect adjacent graphene plates to create denser conductive networks. | 128% | 2296 | Tactile sensor for monitoring human motions | ^[17]^ |
| 18 | Liquid-metal (EGaIn) circuits on ZnO NPs  anchored microfibers | Bulk-state LM film will break up into meshes  on the fiber networks | >1000% | <1 | Personal health management, including moisture wicking,  visualized Joule heating, and human motion/biopotential monitoring | ^[18]^ |
| 19 | Polyurethane (TPU)/multi-walled carbon nanotube (MWCNT) | Propagation of cracks | 70% | 630 | Strain/pressure dual-mode tactile sensor | ^[19]^ |
| 20 | Carbon black (CB)-  coated nylon, silver-plated nylon and elastic spandex yarns-based core-sheath sensing yarns | Space between the double-helical sensing yarns is reduced, resulting increased capacitance. | 70% | 0.854 | Motion monitoring and thermotherapy for human joint injuries | ^[20]^ |
| 21 | (LM@Cu) on flexible substrates | Propagation of Cracks and  wrinkles | 600% | 47 | Stretchable luminous  wristbands, flexible wearable strain sensors, and “visible” thermotherapy panels for relieving aching joints | ^[21]^ |
| 22 | PAA–LM/rGO hydrogels | The changes in closed pores | 1000% | 9.86 | Sensing application in various physical stimuli, such as temperature, solvent, and even vacuum | ^[22]^ |
| 23 | MXene/PPy@PDMS sponge | Pores inside the sponge gradually  shrink and the air inside the sponge is squeezed out by the contact  and mutual stacking of the skeletons | 80% | 1~2 | Human body activity signals, such as radial artery pulse and different joint movements. | ^[23]^ |
| 24 | Biphasic composite comprising of graphene network and liquid metal  (m-GLM) | Crack propagation and bridging, as well as layer slippage | 300% | 281 | Neural network-powered omnidirectional motion, gesture recognition, strain magnitude-adjusted thermotherapy, intuitive thermochromic overheating alerts, and quantitative cervical spine monitoring via wireless sensor arrays. | Our work |

**Reference**

[1] L. T. Yu, J. C. Yeo, R. H. Soon, T. Yeo, H. H. Lee, C. T. Lim, *ACS Appl. Mater. Interfaces* **2018**, 10, 12773.

[2] S. Wan, Z. Zhu, K. Yin, S. Su, H. Bi, T. Xu, H. Zhang, Z. Shi, L. He, L. Sun, *Small Methods* **2018**, 2, 1700374.

[3] R. Guo, H. Wang, G. Chen, B. Yuan, Y. Zhang, J. Liu, *Applied Materials Today* **2020**, 20, 100738.

[4] T. Hu, T. Pan, D. Guo, Y. Xiao, F. Li, M. Gao, Z. Huang, J. Zhu, T. Cheng, Y. Lin, *ACS Nano* **2023**, 17, 22035.

[5] S. Wu, S. Peng, Z. J. Han, H. Zhu, C. H. Wang, *ACS Appl. Mater. Interfaces* **2018**, 10, 36312.

[6] Y. Cai, J. Shen, Z. Dai, X. Zang, Q. Dong, G. Guan, L.-J. Li, W. Huang, X. Dong, *Adv. Mater.* **2017**, 29, 1606411.

[7] Z. X. Zhang, L. Tang, C. Chen, H. T. Yu, H. H. Bai, L. Wang, M. M. Qin, Y. Y. Feng, W. Feng, *J. Mater. Chem. A* **2021**, 9, 10.

[8] L. y. Zhou, J. z. Fu, Q. Gao, P. Zhao, Y. He, *Adv. Funct. Mater.* **2019**, 30.

[9] J. Xu, Z. Wang, J. You, X. Li, M. Li, X. Wu, C. Li, *Chem. Eng. J.* **2020**, 392, 123788.

[10] N. Sheng, P. Ji, M. Zhang, Z. Wu, Q. Liang, S. Chen, H. Wang, *Adv. Electron. Mater.* **2021**, 7.

[11] T. Huang, P. He, R. Wang, S. Yang, J. Sun, X. Xie, G. Ding, *Adv. Funct. Mater.* **2019**, 29, 1903732.

[12] Y. Zhao, S. Zhang, T. Yu, Y. Zhang, G. Ye, H. Cui, C. He, W. Jiang, Y. Zhai, C. Lu, X. Gu, N. Liu, *Nat. Commun.* **2021**, 12, 4880.

[13] Y. Qiao, X. Li, J. Jian, Q. Wu, Y. Wei, H. Shuai, T. Hirtz, Y. Zhi, G. Deng, Y. Wang, G. Gou, J. Xu, T. Cui, H. Tian, Y. Yang, T. L. Ren, *ACS Appl Mater Interfaces* **2020**, 12, 49945.

[14] Y. Lin, Q. Yin, H. Jia, Q. Ji, J. Wang, *Chem. Eng. J.* **2024**, 487.

[15] M. Asaduzzaman, O. Faruk, A. A. Samad, H. Kim, M. S. Reza, Y. Lee, J. Y. Park, *Advanced Functional Materials* **2024**, 34.

[16] V. Sankar, A. Nambi, V. N. Bhat, D. Sethy, K. Balasubramaniam, S. Das, M. Guha, R. Sundara, *ACS Omega* **2020**, 5, 12682.

[17] A. C. Haridas, S. Sharma, K. Naskar, T. Mondal, *ACS Appl Mater Interfaces* **2023**, 15, 17279.

[18] J. Dong, Y. Peng, X. Nie, L. Li, C. Zhang, F. Lai, G. He, P. Ma, Q. Wei, Y. Huang, T. Liu, *Advanced Functional Materials* **2022**, 32.

[19] Z. Yang, Q. Zhang, Q. Luo, J. Shen, C. Yang, D. Li, X. Yin, X. Liu, *Materials & Design* **2025**, 254.

[20] Y. Liu, D. Xu, C. Ge, C. Gao, Y. Wei, Z. Chen, Z. Su, K. Liu, W. Xu, J. Fang, *Adv Sci (Weinh)* **2024**, 11, e2305312.

[21] J. R. Zhang, A. Li, X. L. Li, Y. B. Zhao, J. S. Sun, X. X. Guo, W. Wang, J. Liu, Y. L. Zhang, D. D. Han, *ACS Appl Mater Interfaces* **2025**, 17, 18940.

[22] Z. Zhang, L. Tang, C. Chen, H. Yu, H. Bai, L. Wang, M. Qin, Y. Feng, W. Feng, *J Mater Chem A* **2021**, 9, 875.

[23] H. Xia, L. Wang, H. Zhang, Z. Wang, L. Zhu, H. Cai, Y. Ma, Z. Yang, D. Zhang, *Microsyst Nanoeng* **2023**, 9, 155.
